# Exogenous Pi supplementation improved the salt tolerance of maize (*Zea mays* L.) by promoting Na^+^ exclusion

**DOI:** 10.1038/s41598-018-34320-y

**Published:** 2018-11-01

**Authors:** Yanling Sun, Chunhua Mu, Hongxia Zheng, Shouping Lu, Hua Zhang, Xuecai Zhang, Xia Liu

**Affiliations:** 1 Maize Research Institute, Shandong Academy of Agricultural Sciences/National Engineering Laboratory of Wheat and Maize/Key Laboratory of Biology and Genetic Improvement of Maize in Northern Yellow-huai River Plain, Ministry of Agriculture, Jinan, 250100 Shandong China; 2 College of Life Sciences, Shandong University, Jinan, 250100 Shandong China; 3 International Maize and Wheat Improvement Center (CIMMYT), El Batan, Mexico; 4 College of Life Sciences, Shandong Normal University, Jinan, 250000 Shandong China

## Abstract

The mechanism of phosphate (Pi)-mediated salt tolerance in maize is poorly understood. In this study, the effects of Pi (H_2_PO_4_^−^) on the salt tolerance of two contrasting genotypes was investigated in a pot experiment. We discovered that the application of 3 mM Pi could alleviate salt injury caused by 200 mM NaCl. High amounts of compatible solutes and low amounts of reactive oxygen species (ROS) were also observed under Pi application. Consistent with the increased tolerance, the total number of roots and the growth of shoots increased to relieve salt stress. This phenomenon could be associated with the observed increased expression of nitrate transporters. Furthermore, the seedlings presented a negative relationship between sodium (Na^+^) and Pi (low Na^+^ content and high Pi content), which is related to the genes *ZmNHX*1, *ZmPHT1;8*, and *ZmPHT1;9*, indicating that the exclusion of Na^+^ was promoted by high Pi uptake. However, high Na^+^ and low potassium (K^+^) efflux were detected in the roots, and these were positively correlated with two K^+^ transporters. These observations indicate that Na^+^ exclusion was directly induced by high K^+^ retention rather than Pi absorption. We conclude that maize salt tolerance increased in response to Pi application by promoting Na^+^ exclusion.

## Introduction

Salinity stress is a major environmental factor that constrains plant growth and crop productivity^1,2^. Salinity has degraded 20% of the world’s irrigated land, and almost 7% of the cultivated land area in China is either at risk or already degraded by salinity^3^. Maize is one of the most important food crop species, and maize production has been increasing^4^; however, the growth of maize, a moderately salt-sensitive plant species, is severely limited under salinity, especially during the seedling stage^5^. Therefore, effective methods must be developed to improve the salt tolerance of maize seedlings.

Salinity severely impedes the shoot growth of maize, mainly by reducing the elongation of cells and/or the rate of cell elongation^6,7^. Salt stress may induce membrane damage and accelerate leaf abscission in maize, consequently leading to the accumulation of oxidizing substances and a decline in photosynthesis^7–9^. Moreover, studies have suggested that sodium (Na^+^) plays a major role in salinity toxicity in plants^7^. Plants adapt to salinity stress by employing various measures, including the exclusion of Na^+^ and/or its sequestration into the vacuole, the accumulation of compatible solutes, and the effective scavenging of reactive oxygen species (ROS)^10–13^. In maize, several adaptations also occur^14,15^. Especially for maize seedlings, salt tolerance is associated with the ability to remove Na^+^ and retain potassium (K^+^), and the growth reduction might be due to a decrease in K^+^ levels in the leaves or roots^16,17^. In addition, plants acclimated to salinity can accumulate more Na^+^ in the shoots, and improved vacuolar Na^+^ sequestration plays a vital role in this process^18^.

The exogenous application of many compounds, such as nitric oxide^19^, K^+ ^^20^, brassinolide^21^, spermine^22^, silicon (Si)^23^, melatonin^24^, selenium (Se)^25^, and allantoin^26^, improves plant salt tolerance. Phosphorus (P) plays important roles in various plant growth and metabolic processes and is primarily acquired in its inorganic phosphate (Pi) form as HPO_4_^2−^ or H_2_PO_4_^−^ ions via multiple Pi transporters^27,28^. Improving a plant’s Pi level alleviates premature leaf senescence and improves resistance to cold, disease and lodging^29^. However, Pi exists at extremely low concentrations in soils, especially those with high salinity levels^30^. Exogenous Pi supplementation can influence plant salt tolerance, but this theory is still inconclusive. For instance, Pi application can improve the salt tolerance of violet^31^, cucumber and pepper^32^ but aggravates the salt-induced injury of melon and soybean^33,34^; furthermore, Pi application has no effect on the salt tolerance of lucerne or tepary bean^35,36^.

Na^+^ uptake is enhanced by exogenous Pi application and is associated with reduced expression of *Glycine max Salinity Overly Sensitive 1* (*GmSOS1*) and *Glycine max Cyclic Nucleotide Gated Channel* (*GmCNGC*) in soybean cultivars^34^. In cucumber and pepper cultivars, supplementary P and K reduce high salt stress-induced cell membrane permeability^32^. Exposure to 100 mM salt (10 NaCl:1 CaCl_2_) and high Pi increased both the uptake of Pi and cytoplasmic P in maize seedlings^37^. However, the interaction between salt and Pi and the mechanism underlying the effects of Pi on the salt tolerance of plants are still unclear and need to be further elucidated.

In this study, two contrasting genotypes, namely, QXN233, which is low-Pi (LP) tolerant, and QXH0121, which is LP sensitive^38^, were selected to investigate whether exogenous Pi supply could improve plant salt tolerance. The influences of exogenous Pi on the growth of maize seedlings under salt stress were evaluated, and a physiological response was detected. Furthermore, the molecular mechanism of Pi-mediated salt tolerance in maize was analyzed by transcriptomic and proteomic analyses.

## Results

### Exogenous Pi supplementation improved the salt tolerance of maize genotypes

**Figure 1:**
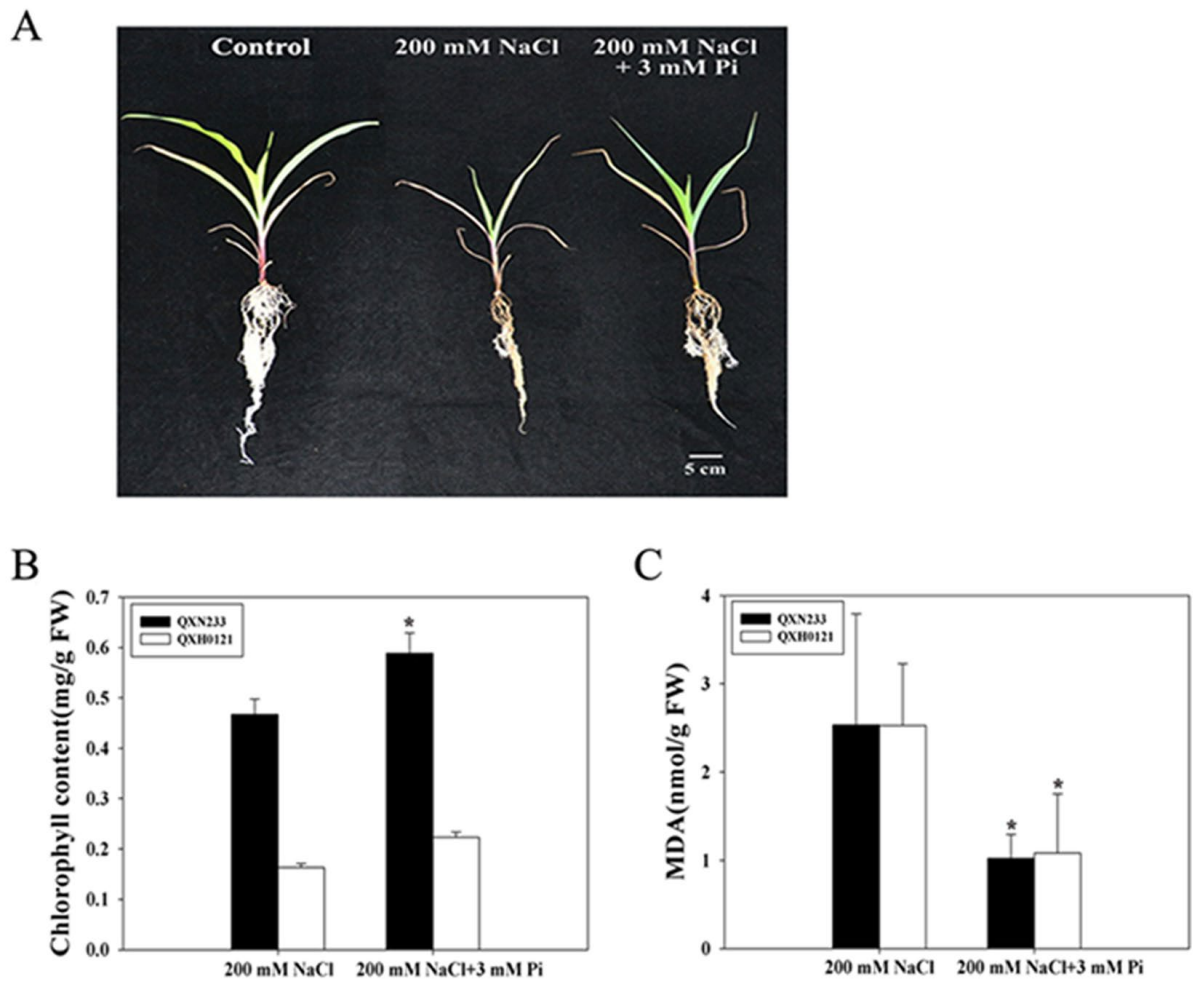
Phenotypic response (**A**) and changes in the contents of chlorophyll (**B**) and MDA (**C**) in QXN233 under NaCl with/without Pi. The bar = 5 cm. The values represent the means ± SEs (n = 3, *P < 0.05).

The maize genotype QXN233 was found to be salt sensitive (Fig. S1) in a preliminary study involving a full factorial combination of various NaCl and Pi concentrations. Under 200 mM NaCl stress, QXN233 had greater numbers of roots when treated with 3 mM Pi than with 11 mM Pi (Figs S2, S3). QXH0121 exhibited similar trends (Figs S4, S5). Quartz sand experiments confirmed that QXN233 grew considerably well; the height, leaf width and length, and fresh weights (FWs) all increased under salt stress when treated with 3 mM Pi (Fig. 1A; Table 1). In addition, increased chlorophyll content and decreased chlorophyll and malondialdehyde (MDA) content were both observed in the leaves of QXN233 and QXH0121, indicating that cell damage become slight due to Pi application under the salt stress conditions (Fig. 1B,C). In summary, these results showed that 3 mM Pi application could strongly alleviate the salt injury caused by 200 mM NaCl in maize genotypes.

**Table 1:** Quantitative analyses of plant height and both the width and length of the longest leaf as well as the FWs of the leaves and roots of QXN233 plants in the different treatment groups after 20 days, as assessed via a quartz sand pot experiment.

QXN233	plant height (cm)	width of the longest leaf (cm)	length of the longest leaf (cm)	fresh weight of the leaves (g)	fresh weight of the roots (g)
Control	33.0 ± 6.0	3.0 ± 0.1	33.0 ± 4.0	7.6 ± 0.5	5.4 ± 0.3
200 mM NaCl	18.0 ± 7.1	2.05 ± 0.1	28.5 ± 1.4	2.7 ± 0.4	2.9 ± 0.2
200 mM NaCl + 3 mM Pi	23.5 ± 0.7^*^	2.55 ± 0.1^*^	32.0 ± 1.4^*^	5.2 ± 0.8^*^	3.8 ± 0.1^*^

The values represent the means ± SEs (n = 3). The asterisks indicate a significant difference between the 200 mM NaCl and the 200 mM NaCl + 3 mM Pi groups (Student’s *t*-test, *P* < 0.05).

### The Pi-mediated increase in root number contributed to increased salt tolerance

**Figure 2:**
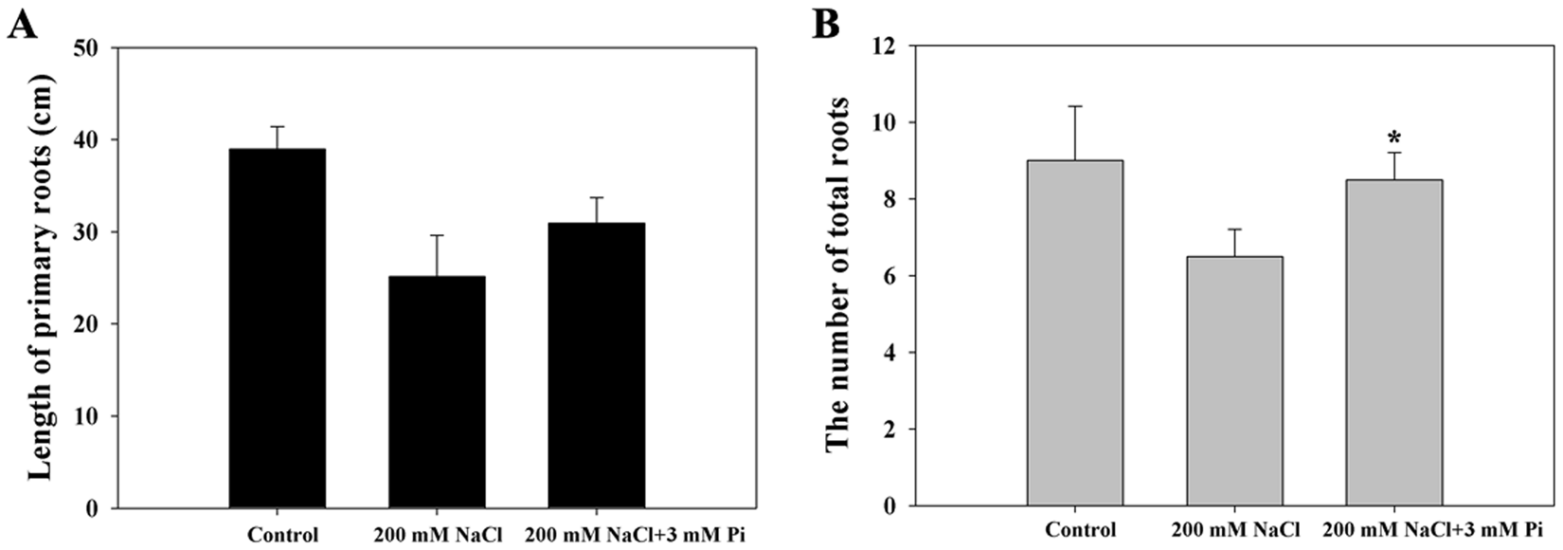
Changes in the length of the primary roots (**A**) and the total number of roots (**B**) of QXN233 under NaCl with/without Pi. The values represent the means ± SEs (n = 3, *P < 0.05).

**Figure 3:**
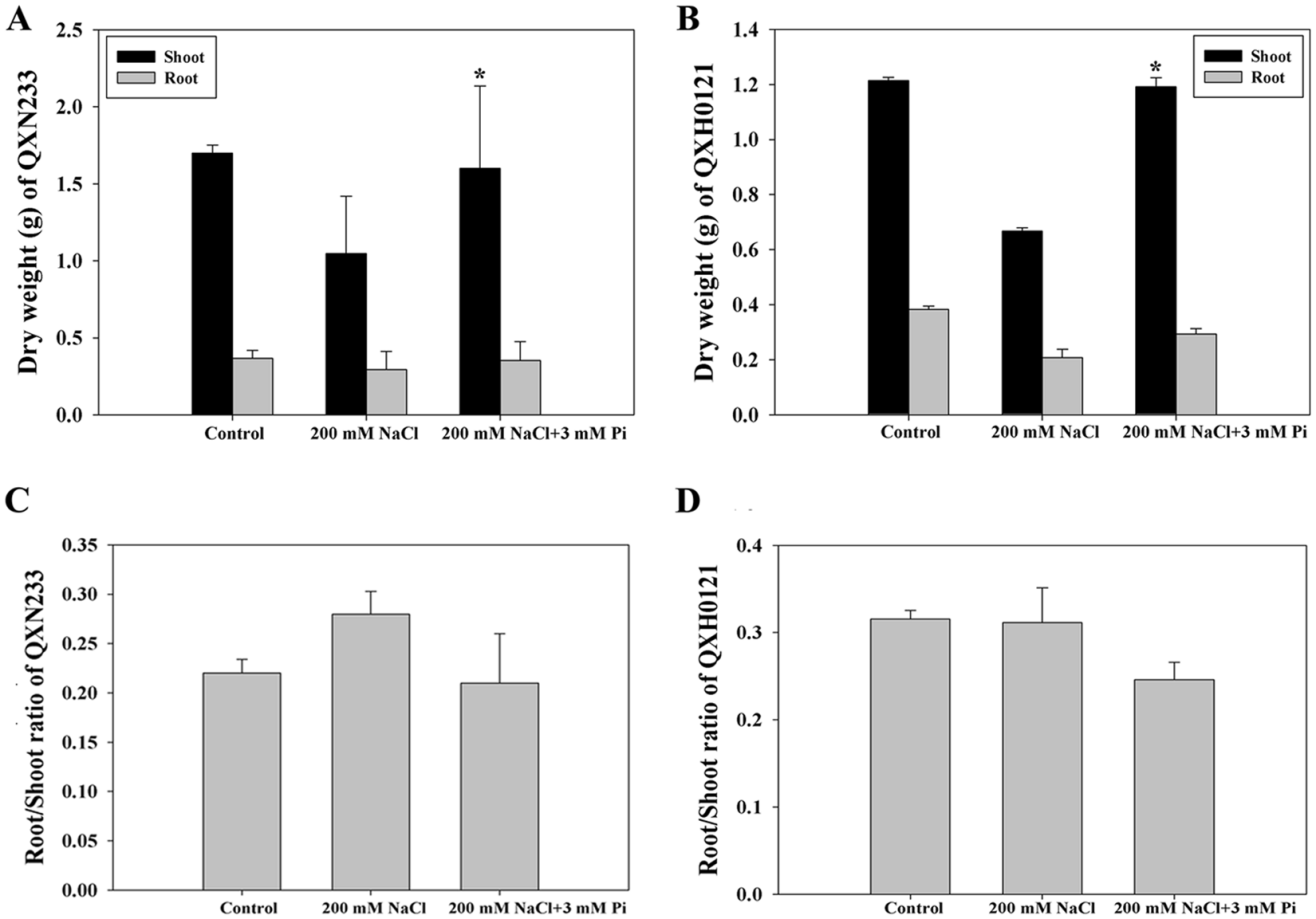
Changes in the DW (**A** or **B**) and root/shoot ratio (**C** or **D**) of maize under NaCl with/without Pi. The values represent the means ± SEs (n = 3, *P < 0.05).

Compared with the control QXN233, the 3 mM Pi-supplemented QXN233 had a greater number of roots (Fig. 2; Fig. S3), indicating that Pi application increased the number of roots rather than the length of the primary roots to mostly improve Pi uptake. The application of Pi resulted in high shoot dry weights (DWs) and a low root/shoot ratio for both QXN233 and QXH0121 (Fig. 3). These results suggested that Pi application could promote the growth of both the roots and shoots of maize seedlings.

### Physiological basis of the increased salt tolerance of maize in response to exogenous Pi supplementation

**Figure 4:**
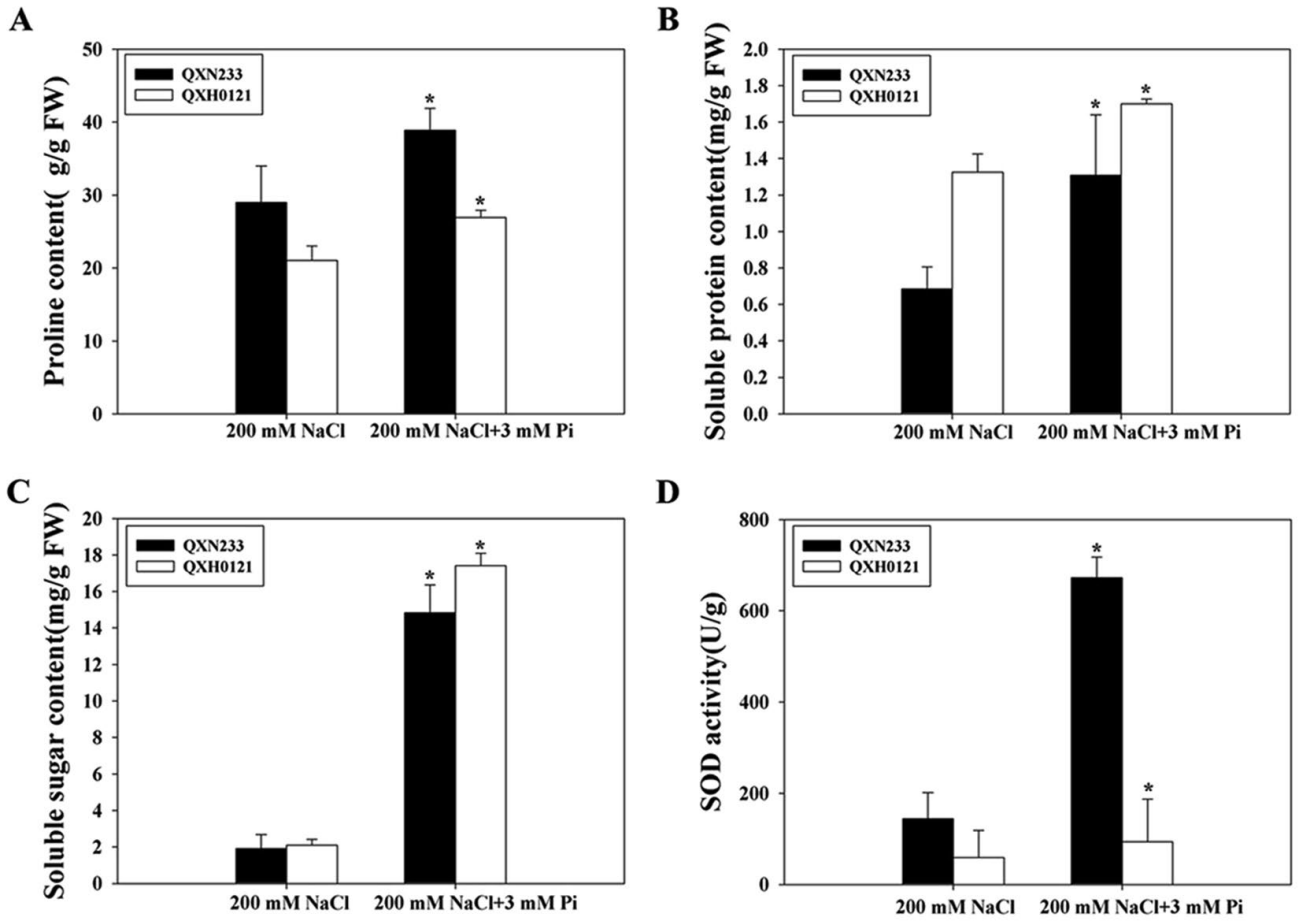
Quantitative analyses of the proline (**A**) soluble protein (**B**) and soluble sugar (**C**) contents and the SOD activity (**D**) in maize under NaCl with/without Pi. The values represent the means ± SEs (n = 3, *P < 0.05).

Under salt stress, the Pi-supplemented QXN233 and QXH0121 seedlings presented high proline, soluble protein, and soluble sugar contents as well as increased superoxide dismutase (SOD) activity (Fig. 4), which could protect the plants from cell damage caused by salt stress. These characteristics contributed to the increased salt tolerance.

### The Na^+^ content was negatively correlated with increased Pi in QXN233

**Figure 5:**
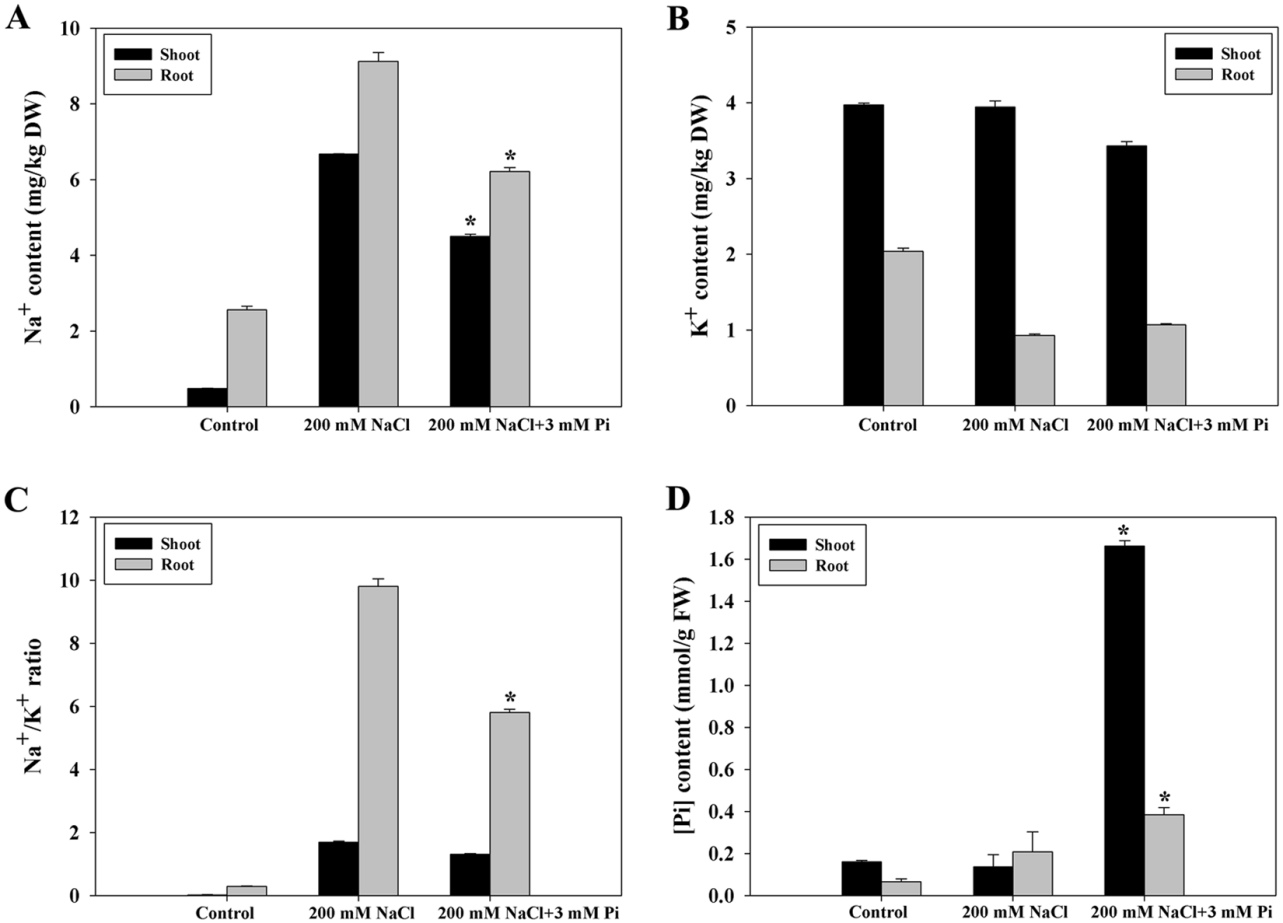
Changes in the Na^+^ (**A**) K^+^ (**B**) and Pi (**D**) contents and the Na^+^/K^+^ ratio (**C**) in QXN233 under NaCl with/without Pi. The values represent the means ± SEs (n = 3, *P < 0.05).

A reduced Na^+^ content and a low Na^+^/K^+^ ratio were observed in the Pi-supplemented QXN233 plants under salt stress (Fig. 5A–C), suggesting that the salt tolerance in improved. In addition, a high Pi content was detected in the Pi-supplemented QXN233 plants (Fig. 5D). Thus, the negative relationship between the Na^+^ and Pi contents indicated that Na^+^ exclusion increased and was mostly related to high Pi absorption.

**Figure 6:**
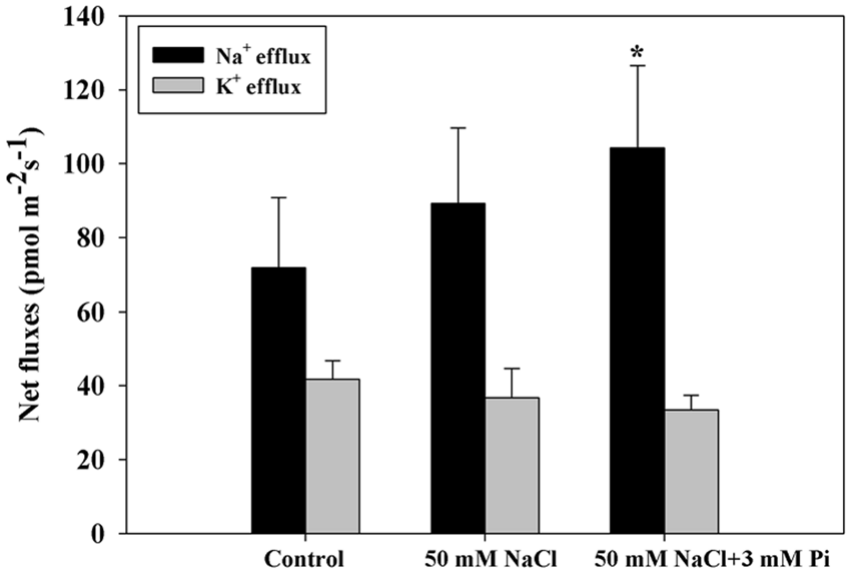
Changes in net Na^+^ and K^+^ fluxes in QXN233. The ion fluxes were measured in the root mature zone of QXN233 under NaCl with/without Pi. The values represent the means ± SEs (n = 3, *P < 0.05).

Furthermore, increased Na^+^ efflux and slightly decreased K^+^ efflux in the roots of Pi-supplemented plants were detected by noninvasive microtest technology (NMT) (Fig. 6), implying that QXN233 exhibited a relatively strong ability to retain K^+^ in response to Pi supplementation. As a result, the Na^+^ exclusion that was associated with Pi absorption was immediately increased by K^+^ retention.

### Expression profiles of relevant genes in QXN233 under high NaCl and Pi conditions

**Figure 7:**
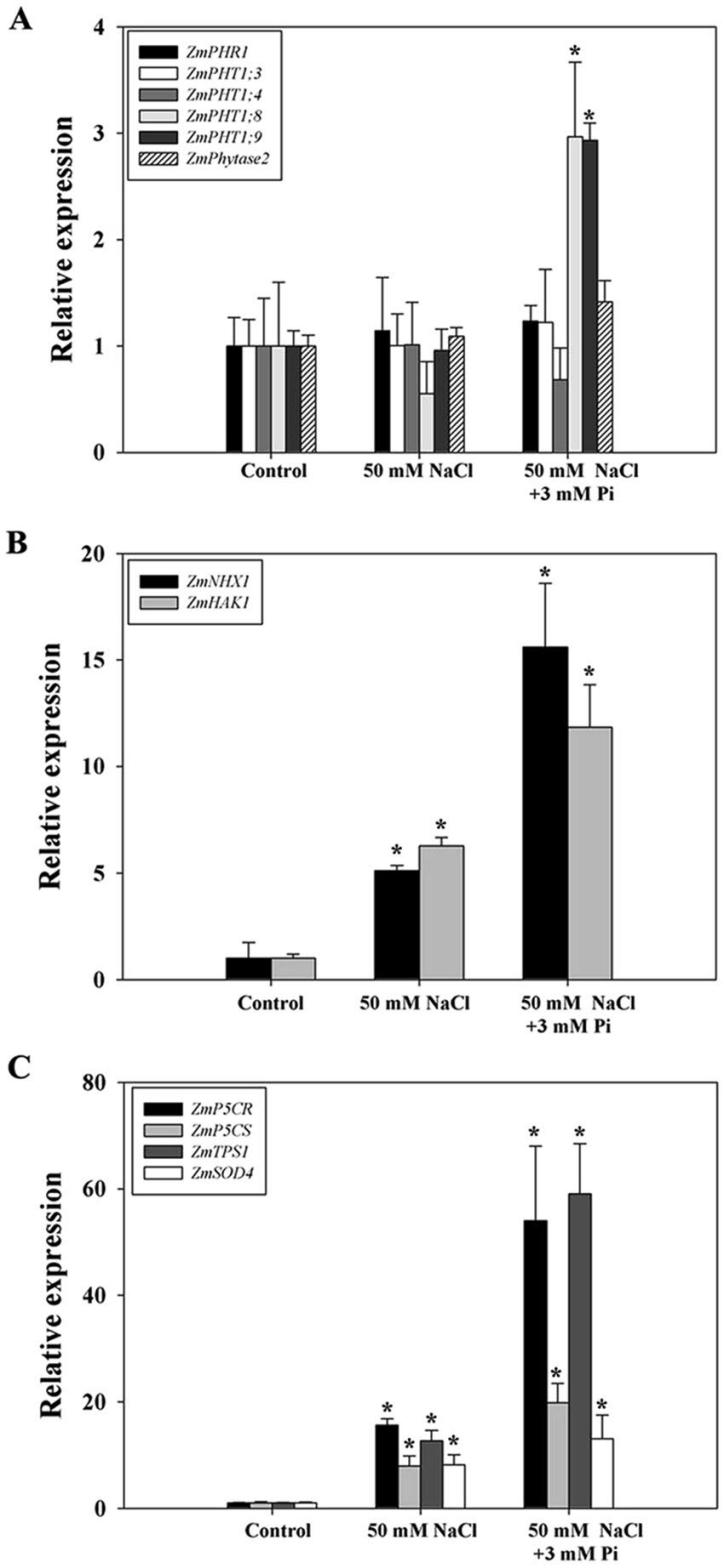
Expression analysis of *ZmPHR1*, *ZmPHT1;3*, *ZmPHT1;4*, *ZmPHT1;8* and *ZmPHT1;9* (**A**); *ZmNHX1* and *ZmHAK1* (**B**); and *ZmP5CR*, *ZmP5CS*, *ZmTPS1* and *ZmSOD4* (**C**) in QXN233 under NaCl with/without Pi. The values represent the means ± SEs (n = 3, ^*^P < 0.05).

In this study, we focused on genes associated with ion transport and stress responses. The results showed that the expression levels of *ZmPHT1;8* and *ZmPHT1;9* were upregulated significantly in the Pi-supplemented plants (Fig. 7A). Moreover, the expression levels of *ZmNHX1* and *ZmHAK1* increased both in the NaCl-treated and Pi-supplemented plants (Fig. 7B), corresponding to the measured Na^+^, K^+^ and Pi ion contents (Fig. 5). Thus, these transporters could play important roles in ion homeostasis to improve QXN233 salt tolerance.

*ZmP5CR*, *ZmP5CS*, *ZmTPS1* and *ZmSOD4* were all highly expressed in the Pi-supplemented plants, especially *ZmP5CR* and *ZmTPS1* (Fig. 7C). According to the above results concerning compatibility solutes and SOD activity (Fig. 4), increased osmotic adjustment and ROS scavenging were responsible for the increased salt tolerance of QXN233.

### Transcriptomic analysis of Pi-mediated gene expression profiles in QXN233 under NaCl stress

Transcriptomic analyses were performed on Pi-treated and non-Pi-treated plants. The results showed that 1140 differentially expressed genes (DEGs) were identified in the leaves (399 upregulated and 147 downregulated), and 1724 DEGs were identified in the roots (827 upregulated and 897 downregulated) (Fig. S6; Table S1). These DEGs were involved in metabolic pathways, plant growth, transcriptional regulation, ion transport, redox homeostasis, stress responses, and phytohormone regulation (Tables S2, S3). Moreover, Gene Ontology (GO) enrichment analysis revealed that the DEGs in the leaf chloroplasts and plastids (Fig. S7A) are involved mainly in biological and metabolic processes and play important roles in photosynthesis and metabolism (Fig. S7A). Additionally, the GO analysis revealed that the DEGs in the extracellular region of the roots are involved mostly in the oxidation-reduction process (Fig. S7B), implying that these DEGs play a positive role in ion homeostasis and ROS detoxification. Kyoto Encyclopedia of Genes and Genomes (KEGG) pathway analysis revealed that the DEGs in the leaves and roots participated mostly in the metabolic pathways and biosynthesis of secondary metabolites (Fig. S8), suggesting that Pi application can promote the growth of maize seedlings under salt stress.

**Figure 8:**
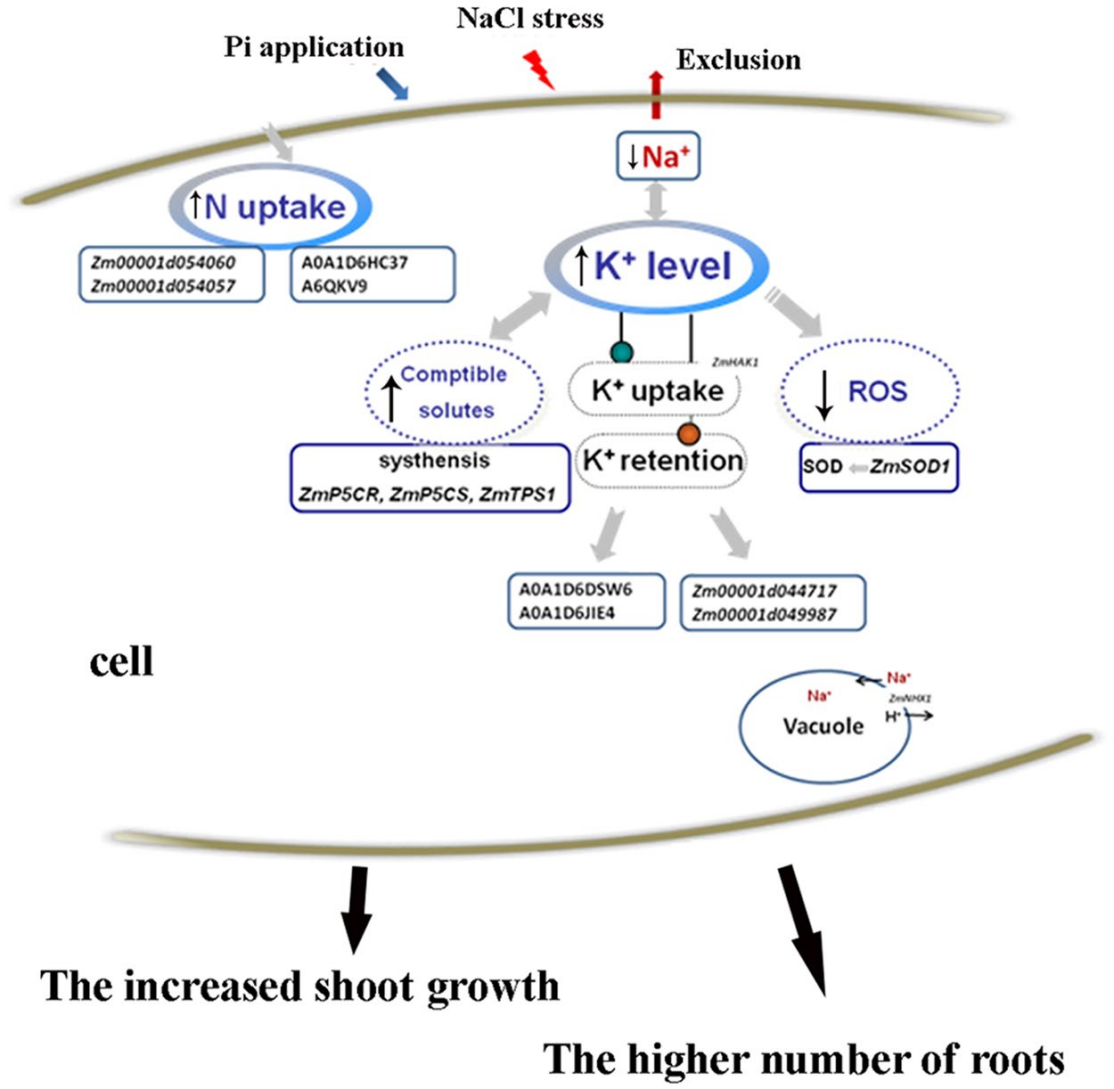
Schematic representation of the mechanism of Pi-mediated salt tolerance in maize. Na^+^ exclusion, which is related to Pi influx, is directly induced by K^+^ retention in plants, and the elevated levels of compatible solutes and reduced ROS contributed to the increased tolerance of maize. Additionally, K^+^ and N uptake, which is associated with relevant transporters, led to the relatively high number of total roots and increased shoot growth to alleviate salt injury. The arrows (↑ or ↓) represent the positive and passive roles of Pi, respectively.

Compared with that in the leaves of the salt-treated plants, the expression of some ion transporters, including *Zm00001d011013* (Calcium pump1), *Zm00001d025831* (Ammonium transporter) and *Zm00001d019936* (Heavy metal-associated domain-containing protein), in the leaves of the Pi-supplemented plants decreased (Table 2; Table S2). Nevertheless, in the roots, the expression of two K^+^ transporters, *Zm00001d044717* (Potassium outward rectifying channel) and *Zm00001d049987* (Potassium transporter), was upregulated in the Pi-supplemented plants (Table 2; Table S2), indicating that K^+^ transport in the roots increased in response to Pi application. The expression levels of two high-affinity nitrate transporters, *Zm00001d054060* and *Zm00001d054057*, were upregulated fourfold in the Pi-supplemented plants compared with the non-Pi-supplemented plants (Table 2; Table S2), suggesting that nitrate transport in the roots increased in response to Pi application. However, no Pi transporters were identified at the transcript level. The long-term treatment time had probably stabilized for Pi transport. These results indicated that Pi application promoted the uptake of K^+^ and nitrate in the roots and could slow plant growth. The K^+^ acquisition further accelerated the exclusion of Na^+^ to reduce the Na^+^ toxicity under salt stress (Fig. 8).

**Table 2:** Transporters that are differentially expressed between the 200 mM NaCl and the 200 mM NaCl + 3 mM Pi groups, as determined by transcriptomic analysis (the fold change is |log_2_(200 mM NaCl + 3 mM Pi/200 mM NaCl)| > 1.0, and P < 0.05).

Tissues	Gene ID	Gene description	Fold change	P value
Leaves	*Zm00001d011013*	Calcium pump1	−1.118	8.41E-05
	*Zm00001d025831*	Ammonium transporter	−2.818	2.14E-05
Roots	*Zm00001d054060*	High-affinity nitrate transporter	5.864	3.75E-07
	*Zm00001d054057*	High-affinity nitrate transporter	4.883	0.000145
	*Zm00001d044717*	Potassium outward rectifying channel	2.385	3.82E-07
	*Zm00001d049987*	Potassium transporter	1.91	4.71E-11
	*Zm00001d034782*	Ammonium transporter	1.737	1.74E-17

### Proteomic analyses of Pi-mediated protein expression profiles in QXN233 under NaCl stress

Proteomic analyses were performed on Pi-treated and non-Pi-treated plants. The results revealed 365 (169 upregulated and 196 downregulated) differentially expressed proteins (DEPs) in the leaves and 259 (128 upregulated and 131 downregulated) DEPs in the roots (*P* < 0.05) (Fig. S9). These DEPs are involved in metabolism, plant growth, transcriptional regulation, ion transport, stress responses, and phytohormone regulation (Tables S4, S5). GO analysis revealed that the DEPs in the leaves and roots are grouped mostly into metabolic processes and cellular processes and are located within the cell and cellular components (Fig. S10). These findings suggest that these proteins participate mainly in metabolic pathways and are in agreement with the results of the transcriptomic analysis. Furthermore, KEGG pathway analysis revealed that the DEPs in both the leaves and roots are involved primarily with ribosomes (Fig. S11) and nitrogen (N) metabolism in the roots, indicating that the roles of Pi in association with ribosomes and N metabolism were strengthened by Pi applications (Fig. S11).

The expression levels of two K^+^ efflux antiporters in the leaves, A0A1D6DSW6 and A0A1D6JIE4, were downregulated under salt stress in the Pi-supplemented plants compared to the non-Pi-supplemented plants (Table 2; Table S4), indicating that the K^+^ efflux in QXN233 leaves decreased under Pi application. Corresponding to the results of the transcriptomic analysis of QXN233 roots, the expression of both A0A1D6HC37 (high-affinity nitrate transporter) and A6QKV9 (putative high-affinity nitrate transporter) was upregulated in the Pi-supplemented plants (Table 2; Table S4), suggesting that the promotion of Pi strengthened the nitrate requirement. However, the expression of both Q6GUH9 (Phosphate transport protein) in the leaves and C4JC09 (Phosphate transporter protein 9) in the roots was downregulated in the Pi-treated plants (Table 2). The long-term Pi supplementation probably increased the cellular Pi level to near saturation and reduced the expression levels of Pi transporters. Proteomic analysis of QXN233 revealed that the expression of two K^+^ efflux transporters decreased in the leaves, whereas that of two high-affinity nitrate transporters increased in the roots under NaCl and Pi treatment conditions. These findings implied that high K^+^ maintenance and nitrate uptake promoted by Pi application could facilitate plant growth by attenuating salt injury and improving salt tolerance (Fig. 8).

## Discussion

The seedling stage is vital for maize growth and is sensitive to various detrimental environmental conditions^5^. In particular, salt stress severely impedes the growth and production of maize^39^. Thus, effective measures must be taken to reduce salt injury at the seedling stage. Exogenous application of substances such as brassinolide^21^, spermine^22^, Se^25^, and allantoin^26^ can improve the salt tolerance of plants. For instance, high K^+^ concentrations were maintained in tomato (*Solanum lycopersicum*) under salt stress^40^, and a relatively low K^+^ efflux improved the salt tolerance of barley by exogenous proline and glycine betaine supplementation^41,42^. Exogenous applications of Se, an essential micronutrient, were recently shown to alleviate salt stress in maize via the improvement of photosynthetic capacity, the activities of antioxidant enzymes and the compartmentalization of Na^+^. Similarly, P, an essential macronutrient, was measured in the present study, and the results showed that Pi (3 mM Pi) in Hoagland’s nutrient solution alleviated the salt injury of maize seedlings under 200 mM NaCl stress (Fig. 1; Figs S2–S5), which might be related to the accumulation of compatible solutes, reduced ROS levels and increased Na^+^ exclusion. This information can be applied in practice and could serve as the basis for the agricultural practice of applying chemical fertilizers that are supplemented with Pi, as this compound has no toxic effects on the environment. In addition, Pi supply could significantly reduce the Na^+^ levels in the shoots and roots (Fig. 5), suggesting that Pi could negatively regulate Na^+^ levels in plant cells. Thus, we speculated that the tolerant genotypes could grow and develop with a relatively low demand for Pi, which suggests that widespread cultivation of salt-tolerant plants would be favorable to coping with the problem of increasingly depleted global sources of P^43^.

Pi applications can help many crops resist various adverse conditions^29^. On the basis of the results of our physiological investigation, we discovered that, compared with the control seedlings, the Pi-supplemented seedlings presented increased plant height, leaf width, leaf length, and shoot and root FW and DW (Table 1; Figs 1–3). However, continuously adding Pi to plants under salt stress is not beneficial. As shown in Fig. S2B, the performance of the plants under NaCl was poorer when combined with 11 mM Pi than with 3 mM Pi, suggesting that oversupplementation could lead to severe osmotic pressure rather than nutritional absorption in plants^44,45^. Similar to Se in maize, Pi stimulates plant growth at low concentrations but is toxic to plant growth and development at high concentrations^25^. As major phenotypic adaptations to improved Pi levels under salt stress, both the total number of roots and the DWs of shoots of the maize seedlings increased, and the root/shoot ratio decreased (Figs 2, 3), revealing that high Pi uptake promoted both the root architecture and shoot growth of plants under salt stress^27^; moreover, as an adaptive measure of plants to maintain their functional equilibrium, a low root/shoot ratio is consistent with the results of many studies on other plant species^16,46^. Salinity severely impedes shoot growth, mainly by reducing the number of elongating cells and/or the rate of cell elongation^6,7^. Pi supplementation probably corrected these deficiencies. The expression of some cell-elongating relevant genes/proteins, such as the *Alpha-expansin 1* (*Zm00001d043047*), *Beta-expansin 1a* (*Zm00001d047096*) and *Cell number regulator 9* (*Zm00001d012899*) genes as well as the Alpha-expansin 5 (Q94KT3), Cellulose synthase (A0A077D360), and Cell division cycle (A0A1D6GQ42) proteins, was upregulated in this study (Tables S2, S4), providing evidence that Pi supply accelerates the growth of maize seedlings.

Plant responses to salinity occur in two phases: a rapid, osmotic phase and a slower, ionic phase^7^. Compatible solutes usually accumulate in the cell cytosol and organelles to balance the osmotic pressure of the ions in the vacuole, and antioxidative enzymes are active to mitigate oxidative damage initiated by ROS^7,9^. In this study, under salt stress, the Pi-supplemented plants presented high chlorophyll contents, low MDA levels, and elevated compatible solutes and SOD activity (Figs 1, 4), which yielded important tolerance indexes. Specifically, high proline accumulation can positively reduce K^+^ efflux and maintain K^+^ levels in plant cells^40,41^, corresponding to elevated expression levels of *ZmP5CR* and *ZmP5CS* (Figs 4A and 7C). These results indicated that an improved ability for osmotic adjustment and ROS scavenging were key factors in the improved salt tolerance of QXN233, which was also supported by the results of previous studies^47,48^. A negative correlation between the Na^+^ and Pi contents (low Na^+^ content and high Pi content) was observed in the Pi-supplemented plants under salt stress (Fig. 5), implying that Na^+^ transport was negatively regulated by exogenous Pi influx. However, given that Na^+^ is a positive ion and Pi is a negative ion, whether Na^+^ contents in plants are regulated directly by Pi absorption is still unclear. Measurements of Pi ion flux via NMT would provide evidence for this question in the future. Moreover, a high Na^+^ efflux and slightly low K^+^ efflux in the maize roots was detected via NMT (Fig. 6), indicating that K^+^ retention improved the salt tolerance of maize, as supported by our previous findings in *Arabidopsis*^49,50^. Thus, it was concluded that Na^+^ exclusion in the Pi-supplemented plants was directly strengthened by the high K^+^ retention and was positively associated with sufficient Pi absorption under salt stress. In addition, studies have shown that soybean plants supplied with exogenous Pi presented increased Na^+^ uptake and reduced salt tolerance, which is probably due to the specific physiological structure of soybean plants, which differs from that of maize plants^34^. On the basis of the results of the present study, exogenous Pi application had various effects on the salt tolerance of different plant species, and these effects are related to the different mechanisms of Pi-mediated salt tolerance in those plants^31–34,36^.

Transcriptomic and proteomic analyses both revealed that the DEGs/DEPs in the Pi-supplemented plants under NaCl stress were involved mainly in metabolism and biological processes (Tables S2, S4), suggesting that Pi plays a major role in the growth and metabolism of plants. The elements N, P and K are three essential primary nutrients for plants^51^. In the present study, the data analysis revealed that expression of two high-affinity nitrate transporter genes, *Zm00001d054060* and *Zm00001d054057*, and relevant proteins, A0A1D6HC37 and A6QKV9, was elevated in the roots (Tables 2, 3; Tables S2, S4). This finding indicated that strong nitrate uptake induced by Pi application promoted plant growth. However, the accelerated growth clearly increased the K^+^ demand of the plants. The expression of two K^+^ transporter genes, *Zm00001d044717* and *Zm00001d049987*, increased in the roots (Table 2; Table S2), whereas that of the K^+^ efflux transporter proteins A0A1D6DSW6 and A0A1D6JIE4 decreased in the leaves (Table 3; Table S4), implying that a low K^+^ loss occurred in the Pi-supplemented plants under salt stress. This finding was supported by a slightly decreased K^+^ efflux in the roots of the Pi-supplemented plants (Fig. 6). Reports have suggested that K^+^ competes for the binding sites of Na^+^ in cells because of their similar ion radius^52,53^. An increased Na^+^ efflux was detected in the plant roots, which suggested that the high Na^+^ exclusion was directly caused by high K^+^ retention, contributing to the improved salt tolerance, as described previously^18^. This result provided evidence that the natural varieties of salt-tolerant maize depend on the efficiency of Na^+^ exclusion, which is described in previous reports^17,54^. In addition, when relatively high Na^+^ exclusion occurs, greater amounts of Na^+^ are compartmentalized into the vacuoles, which is supported by the high expression levels of *ZmNHX* detected in the maize roots (Fig. 7B). However, no Na^+^ transporters were identified in our data analysis, probably due to the long-term treatment time of the seedlings. In summary, increased Na^+^ exclusion that is correlated with high Pi absorption could be directly induced by high K^+^ maintenance to mitigate Na^+^ toxicity. Indeed, some nitrate and K^+^ transporters play key roles in reducing plant growth to alleviate salt injury (Fig. 8). Therefore, whether salt tolerance can be strengthened/weakened by the overexpression/RNA interference (RNAi) of these transporter genes in maize is worth exploring.

**Table 3:** Transporters that are differentially expressed between the 200 mM NaCl and the 200 mM NaCl + 3 mM Pi groups, as determined by proteomic analysis (the ratio is |200 Na + 3 Pi/200 Na| > 1.2, and P < 0.05).

Tissues	Accession number	Protein description	Ratio	P value
Leaves	C0P6N0	Calcium load-activated calcium channel	1.589	2.88E-09
	A0A096PXB4	Vacuolar cation/proton exchanger 3	0.632	3.78E-09
	A0A1D6HHV5	Cation/H^+^ antiporter 1	0.726	3.70E-05
	A0A1D6DSW6	K^+^ efflux antiporter 2 chloroplastic	0.748	0.00018
	A0A1D6JIE4	K^+^ efflux antiporter 2 chloroplastic	0.813	0.00726
	Q6GUH9	Phosphate transport protein	0.772	0.00084
Roots	A0A1D6HC37	High-affinity nitrate transporter	1.626	0.011126
	A6QKV9	Putative high-affinity nitrate transporter	1.409	0.034144
	B6TGS2	Calcium load-activated calcium channel	1.344	0.004091
	C4JC09	Phosphate transporter protein 9	0.66	0.046708

In roots, high expression levels of some Na^+^, K^+^ and Pi transporter genes, including *ZmNHX1*, *ZmHAK1*, *ZmPHT1;8*, and *ZmPHT1;9*, in response to Pi were detected at 12 h after salinity treatment and were positively correlated with increased salt tolerance and Pi absorption. The transport of Na^+^ into vacuoles is mediated by a tonoplast Na^+^/H^+^ antiporter encoded by *NHX* genes, which are present in both root and leaf cells^55–57^. In this study, we found that the expression of *ZmNHX* was upregulated in the roots of maize plants exposed to high NaCl concentrations, implying that an increased sequestration of Na^+^ into the vacuoles of root cells occurred; a similar result was reported for Se applications after 24 h of salinity stress^25^. In maize, a salt-sensitive species, the expression of *ZmNHX* is usually very weak unless the plants are exposed to prolonged salinity; thus, the above studies involving Pi and Se applications confirmed this point. These results suggest that Na^+^ compartmentalization plays an important role in roots during the early process of Pi/Se application under salt stress. The transport of Na^+^ into the vacuoles of the roots was likely facilitated by prolonged salinity, which prevented the toxic effect of Na^+^ in the leaves; this idea is supported by previous findings in which the inclusion of Na^+^ in the vacuoles of cortical cells was detected in barley^58^. How to regulate this process in maize is unclear, and the molecular mechanism needs to be further analyzed in the future. In addition, it would be interesting to explore how the overexpression of *ZmNHX* could be used to enhance salt tolerance in maize, similar to achievements made with transgenic *Arabidopsis thaliana* and tomato plants^55,57,59,60^.

## Conclusions

We reveal that the salt tolerance of maize could be improved by exogenous Pi applications at the seedling stage. The increased number of total roots and vigorous growth of shoots were discovered to be phenotypic adaptations, and the highly compatible solutes and reduced ROS contributed to this enhanced tolerance. A reduced Na^+^ content was negatively correlated with an increased Pi level in plants, which was attributed to the strong Na^+^ exclusion and was directly associated with K^+^ retention rather than Pi absorption. The highly expressed *ZmNHX1*, *ZmHAK1*, *ZmPHT1;8*, and *ZmPHT1;9* genes could play important roles in this process. Furthermore, transcriptomic and proteomic analyses of the leaves and roots revealed that some K^+^ and nitrate transporters responded positively to NaCl and Pi stress, suggesting that these transporters are probably key molecular targets of exogenous Pi-mediated salt tolerance in maize. Further study of the biological interactions between Pi absorption and these transporters may provide new insights into the molecular mechanism of Pi-mediated salt tolerance and could lay a foundation for breeding salt-tolerant maize. Whether the salt tolerance of maize cultivars can be improved by exogenous Pi supplementation under high salinity conditions should be further investigated.

## Materials and Methods

### Plant growth and treatment conditions

Two maize genotypes, QXN233 and QXH0121, whose tolerance to long-term LP stress differs (described in our previous study^38^), were selected for the present study. The seeds of these plants were surface sterilized for 10 min with 10% sodium hypochlorite solution, rinsed with sterilized distilled water several times, and then germinated on wet sterilized filter paper in petri dishes at 28 ± 2 °C and 60–70% humidity.

For the field experiment, three-day-old seedlings were transplanted to individual pots that were filled with soil. At the small bell-mouth period, the maize plants were treated with Hoagland’s nutrient solution as previously described^34^, and 200 mM NaCl was added to each pot every 3–5 days. The plants were imaged for two weeks. For the vermiculite pot experiment, three-day-old seedlings that lacked endosperm were planted in individual pots that were filled with vermiculite. A 200 mM NaCl solution with or without Pi (H_2_PO_4_^−^) was then added to the pots when the plants had three fully grown leaves. The treatment solutions were applied to each pot every 2 days.

The NaCl and Pi interaction was examined in the vermiculite pot experiment. To screen for the appropriate Pi for plant resistance to salt stress, salt treatments were divided into four groups: 0 mM NaCl, 200 mM NaCl, 400 mM NaCl and 800 mM NaCl. Each group was also supplemented with a different concentration of Pi: 1 mM Pi (control), 3 mM Pi or 11 mM Pi. At 20 days after treatment, the 3 mM Pi supplementation clearly alleviated the salt damage in the maize seedlings caused by the 200 mM NaCl stress. The plants were harvested, analyzed, and imaged.

Three-day-old seedlings were planted in individual pots filled with quartz sand to confirm the above discovery. At the three-leaf stage, the seedlings were treated with a 200 mM NaCl or a 200 mM NaCl + 3 mM Pi nutrient solution for 20 days, after which they were analyzed and imaged.

### Growth parameters and chlorophyll and MDA contents

The plant height and both the width and length of the longest leaf were measured. The roots were rinsed, spread appropriately, and then scanned with an Epson transparency unit (Epson, Beijing, China). The length of the primary roots and the total number of roots were then analyzed. Afterwards, the roots were dried quickly on filter paper. On the other hand, the leaves were harvested directly. The FWs of the leaves and roots were obtained via a laboratory-style electronic balance. The fresh leaves and roots were heated at 105 °C for 30 min and then dried at 85 °C for 3 days. The stable DWs of the leaves and roots were ultimately obtained.

In the vermiculite pot experiment, seedlings at the three-leaf stage were treated for 30 days with 200 mM NaCl or 200 mM NaCl + 3 mM Pi added to the nutrient solution. Leaf samples were harvested, and their chlorophyll and MDA contents were measured in accordance with previously described methods^50^. Each experiment was replicated three times, and six plants were included in each of the three replicate measurements.

### Proline, soluble sugar, and soluble protein contents and SOD activity

The same treatments were applied as described above for MDA measurements. The proline, soluble sugar, and soluble protein contents and the SOD activity in the leaves were measured as described previously^50^. Six plants were included in each of the three replicate measurements.

### Na^+^, K^+^, and Pi contents

In the vermiculite pot experiment, seedlings at the three-leaf stage were treated for 30 days with 200 mM NaCl or 200 mM NaCl + 3 mM Pi added to the nutrient solution. The Na^+^, K^+^ and Pi contents in the leaves and roots were measured in accordance with previous protocols^38,50^. The results are presented as the means and standard errors (SEs) of the means of three biological replicates.

### Na^+^ and K^+^ fluxes measured via NMTs

Different net Na^+^ and K^+^ fluxes in the root mature zone of the maize seedlings at the three-leaf stage were measured via NMTs as described previously^50^ after 30 h of exposure to 50 mM NaCl or 50 mM NaCl + 3 mM Pi. The steady-state ion flux rates were expressed as the means of the measured points of 6 seedlings. The error bars indicate the SEs.

### Transcriptomic data analyses

Six samples were used per group. The total RNA of the leaves and roots was extracted from three-leaf-stage QXN233 seedlings growing under conditions of 200 mM NaCl or 200 mM NaCl + 3 mM Pi for 30 days by using TRIzol (Invitrogen, USA) according to the manufacturer’s instructions. After the quality and integrity of the total RNA were assessed using a NanoPhotometer^®^ spectrophotometer (Thermo Scientific, USA), mRNA was isolated from the total RNA samples using oligo (DT) magnetic beads to purify the polyA-containing mRNA (Invitrogen, CA, USA). The mRNA was fragmented, and double-stranded cDNA was synthesized. The final cDNA libraries were sequenced via an Illumina High-Seq2000 sequencing system (Novogene Co., Ltd., Beijing, China).

After low-quality raw reads were removed, all the RNA-Seq reads were aligned with the maize B73 RefGen_V4 genomic DNA sequence via TopHat 2.0.12 software and annotated to known genes via a BLAST (2.2.23) analysis (http://blast.ncbi.nlm.nih.gov/Blast.cgi). Genes were considered differentially expressed based on a threshold of |log_2_(fold change)| > 1 and *P* < 0.005, which was based on the assessment of the expected number of fragments per kilobase of transcript sequence per million base pairs sequenced (FPKM) and the false discovery rate (FDR). GO enrichment analysis of three ontologies (biological process, molecular function, and cellular component) and KEGG pathway analysis were also performed.

### Proteomic data analysis

The leaves and roots of QXN233 seedlings at the three-leaf stage were collected from the 200 mM NaCl and 200 mM NaCl + 3 mM Pi treatment groups after 30 days, respectively. Six samples were harvested from each group. The tissue samples were frozen in liquid N, ground, precipitated by trichloroacetic acid (TCA):acetone (1:9), resuspended in SDT buffer (4% sodium dodecyl sulfate [SDS], 100 mM Tris-HCl, 1 mM dithiothreitol [DTT], pH = 7.6), sonicated and then boiled for 15 min. After centrifugation, the supernatant was analyzed via a Bicinchoninic Acid (BCA) Protein Assay Kit (Bio-Rad, USA) and then stored at −80 °C until use. The protein quality of each sample was examined with sodium dodecyl sulfate polyacrylamide gel electrophoresis (SDS-PAGE). The protein was then digested in accordance with the filter-aided sample preparation (FASP) procedure as described previously^61^.

Peptide samples derived from the above treatment groups were labeled using a tandem mass tag (TMT) reagent according to the manufacturer’s instructions (Thermo Fisher Scientific). The TMT-labeled digested samples were divided into 10 fractions with a Pierce high pH reversed-phase fractionation kit (Thermo Scientific) by increasing the acetonitrile step-gradient elution according to the manufacturer’s instructions. Each fraction was reconstituted in 0.1% trifluoroacetic acid (TFA). Nano-liquid chromatography-tandem mass spectrometry (LC-MS/MS) analysis was performed on each prepared fraction, and a Q-exactive mass spectrometer (Thermo Scientific) coupled to Easy nLC (Proxeon Biosystems, now Thermo Fisher Scientific, USA) device was used for LC-MS/MS analysis as previously described^62^.

MS/MS spectra were queried using the MASCOT engine (Matrix Science, London, UK; version 2.2) embedded within Proteome Discoverer 1.4. The Decoy database was used for FDR calculations, and results with an FDR ≤ 0.01 were filtered. The statistical significance of the quantitative data was determined using Student’s *t*-test (n = 3, *P* < 0.05), and the DEPs between the two treatment groups were defined when the following criteria were met: *P* < 0.05 and a ratio > 1.2 (up-/downregulated). On the basis of the results of Fisher’s exact test, GO enrichment of three ontologies (biological process, molecular function, and cellular component) and KEGG pathway enrichment analyses were performed to explore the effects of the DEPs on cell physiological processes.

### Quantitative real-time PCR (qRT-PCR) analysis

The total RNA samples of three-leaf-stage seedlings in the 50 mM NaCl and 50 mM NaCl + 3 mM Pi groups were extracted after 12 h by using an E.Z.N.A. Plant RNA Kit (Omega Bio-tek, USA). cDNA was synthesized with 5×All-in-One RT MasterMix (AccuRT Genomic DNA Removal Kit included) (ABM, Canada) and amplified with an Ultra SYBR Mixture Kit (CWBIO, China) on an ABI 7500 Real-time PCR system (ABI, USA). The *18S* RNA gene was used as an internal control, and the data were analyzed by the 2^−∆∆Ct^ method^38,63^. The genes and primers used in this study are listed in Table S1. Each qRT-PCR result was based on three biological replicates and three technical replicates.

### Data analysis

Statistical analysis was performed using SPSS 16.0 software (SPSS, Chicago, IL, USA). All the data obtained were subjected to ANOVA. The values represent the means ± SEs of three replicates. Significant differences between the two treatments groups were determined by the P value (*means *P* < 0.05) based on Student’s *t*-tests.

## Electronic supplementary material

Supporting Information

Table S3

Table S5

## Data Availability

The authors declare that the data are available.
