# Supplementary material for: Exogenous Pi supplementation improved the salt tolerance of maize (Zea mays L.) by promoting Na+ exclusion
Source: Sci Rep. 2018 Nov 1;8:16203. doi: 10.1038/s41598-018-34320-y (PMC6212588; doi:10.1038/s41598-018-34320-y)
Supplement: Supplementary file 1 — Supporting Information [file 41598_2018_34320_MOESM1_ESM.doc]

Supporting Information

**Article title:** Exogenous Pi supplementation improved the salt tolerance of maize (*Zea mays* L.) by promoting Na^+^ exclusion

**Authors:** Yanling Sun, Chunhua Mu, Hongxia Zheng, Shouping Lu, Hua Zhang, Xuecai Zhang, Xia Liu.

The following Supporting Information is available for this article:

**Figure S1** The phenotypic response of QXN233 (A) and QXH0121 (B) exposed for 2 weeks to 200 mM NaCl by using a field assay. The bar = 10 cm.

**
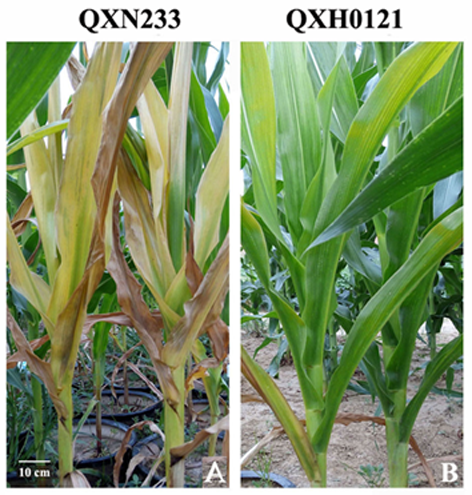
**

**Figure S2** The phenotypic response of the salt sensitive QXN233 exposed for 20 d to 0 mM NaCl (A), 200 mM NaCl (B), 400 mM NaCl (C) and 800 mM NaCl (D) combined with different concentration of external Pi (1 mM, 3 mM or 11 mM Pi), respectively. The bar = 5 cm.

**
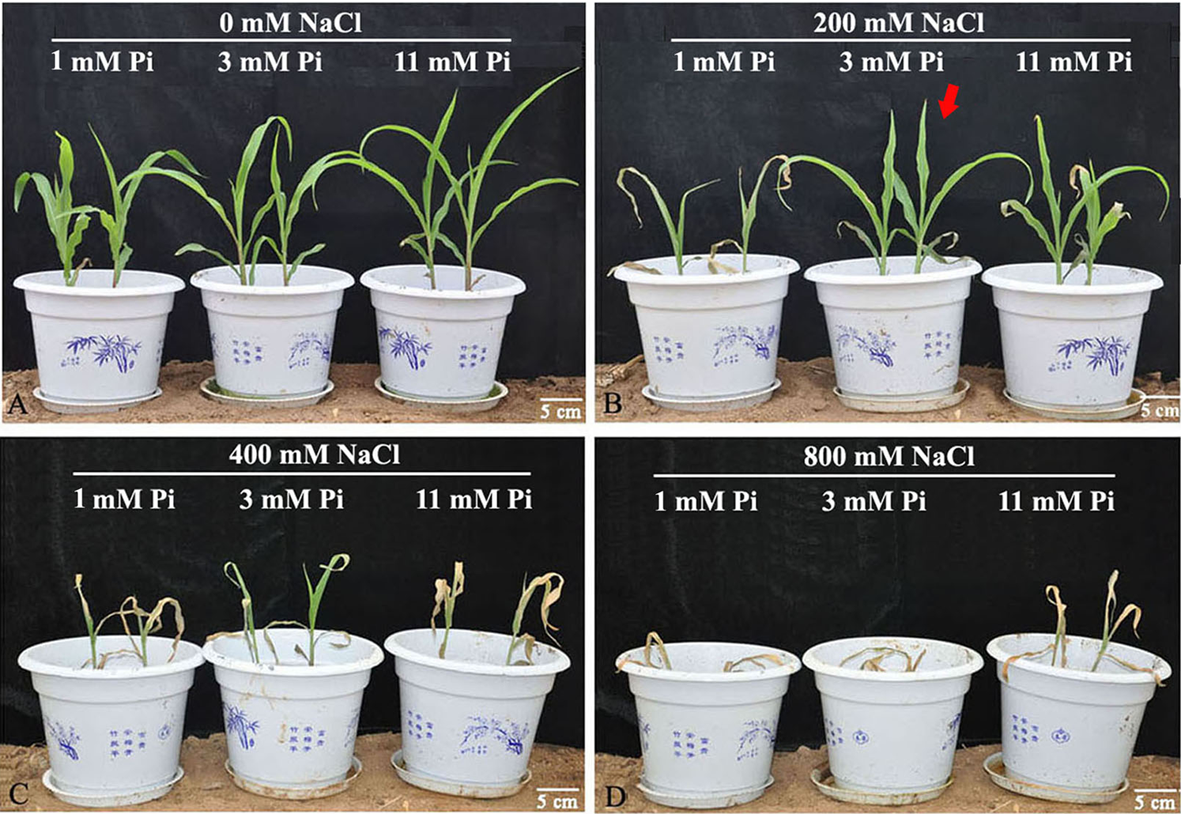
**

**Figure S3** The root response of QXN233 exposed for 20 d to 200 mM NaCl (A) combined with 3 mM Pi (B) and 11 mM Pi (C), respectively. The pictures were scanned with an EPSON Transparency unit (EPSON, Beijing, China). The bar = 2 cm.

**
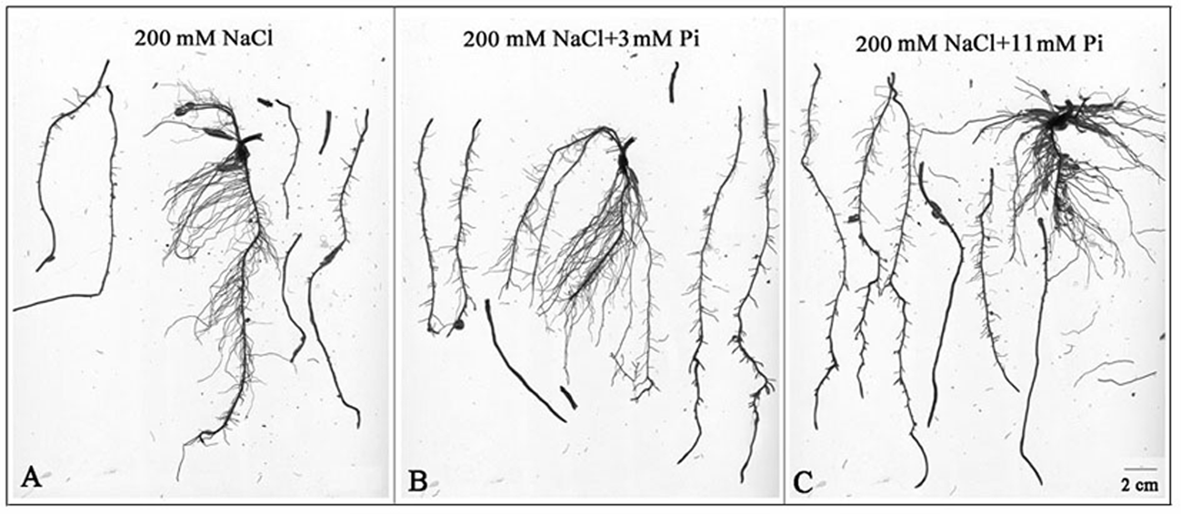
**

**Figure S4** The leaves and roots response of maize genotypes QXN233 (A) and QXH0121 (B) exposed for 25 d to 0 mM NaCl (1), 200 mM NaCl (2), and 200 mM NaClcombined with 3 mM Pi (3), respectively. The bar = 10 cm.

**
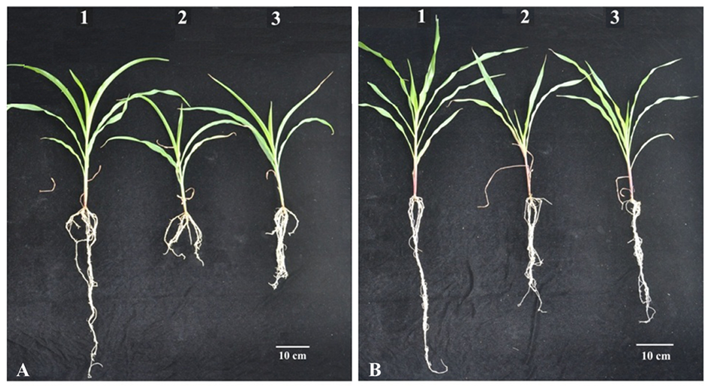
**

**Figure S5** The phenotypic response of maize genotypes QXN233 (A) and QXH0121 (B) exposed for 30 d to 200 mM NaCl combined with 3 mM Pi. The bar = 5 cm.

**
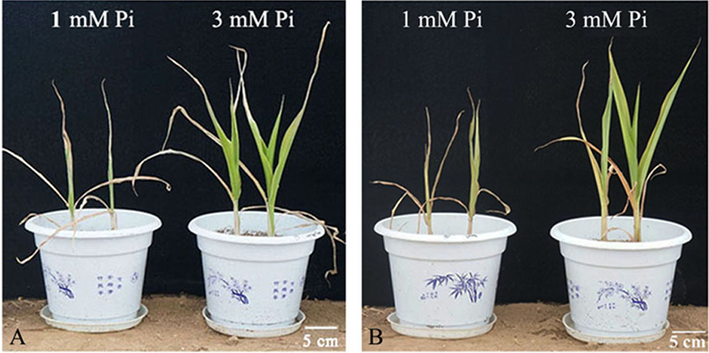
**

**Figure S6** Volcano plot of differentially expressed genes (DEGs) in leaves (A) and roots (B). For every gene, the ratio of expression levels in 200 mM NaCl+3 mM Pi over that in 200 mM NaCl group was plotted against the -Log (p-value). The vertical lines indicate the two fold change threshold, and the horizontal lines indicate p-value = 0.05.

**
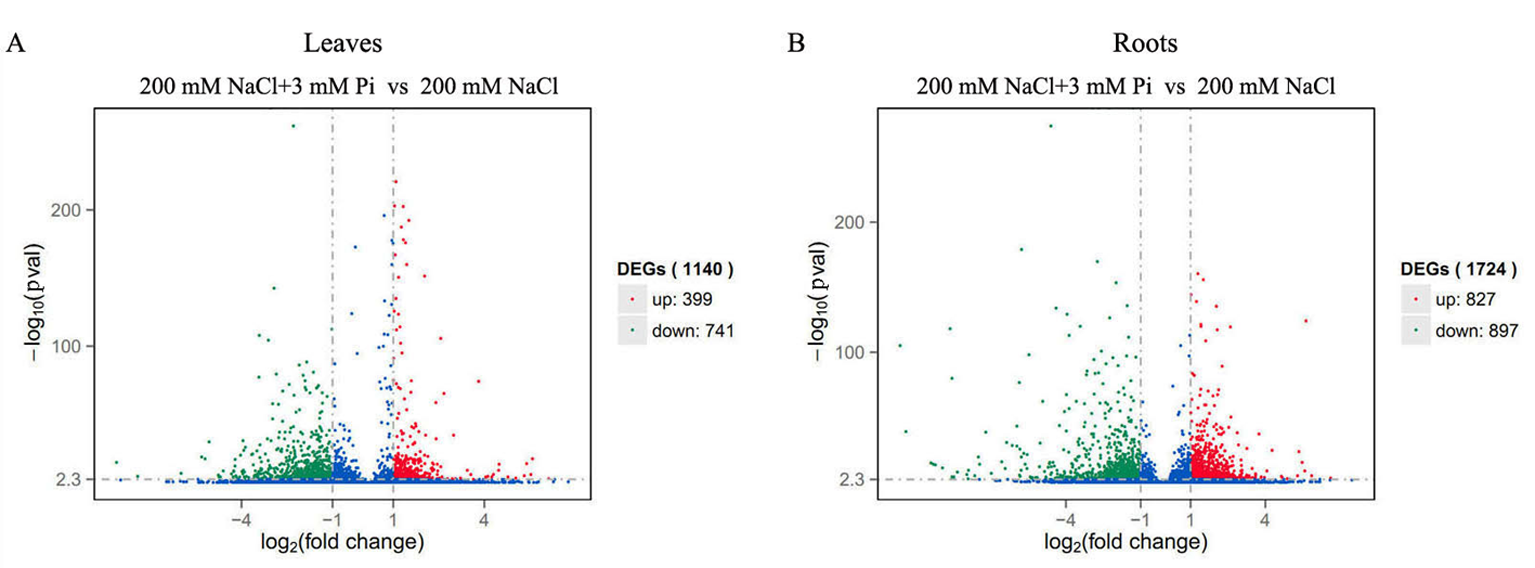
**

**Figure S7** Gene ontology (GO) categories of DEGs based on GO enrichment analysis in leaves (A) and roots (B) between 200 mM NaCl and 200 mM NaCl+3 mM Pi group.

**
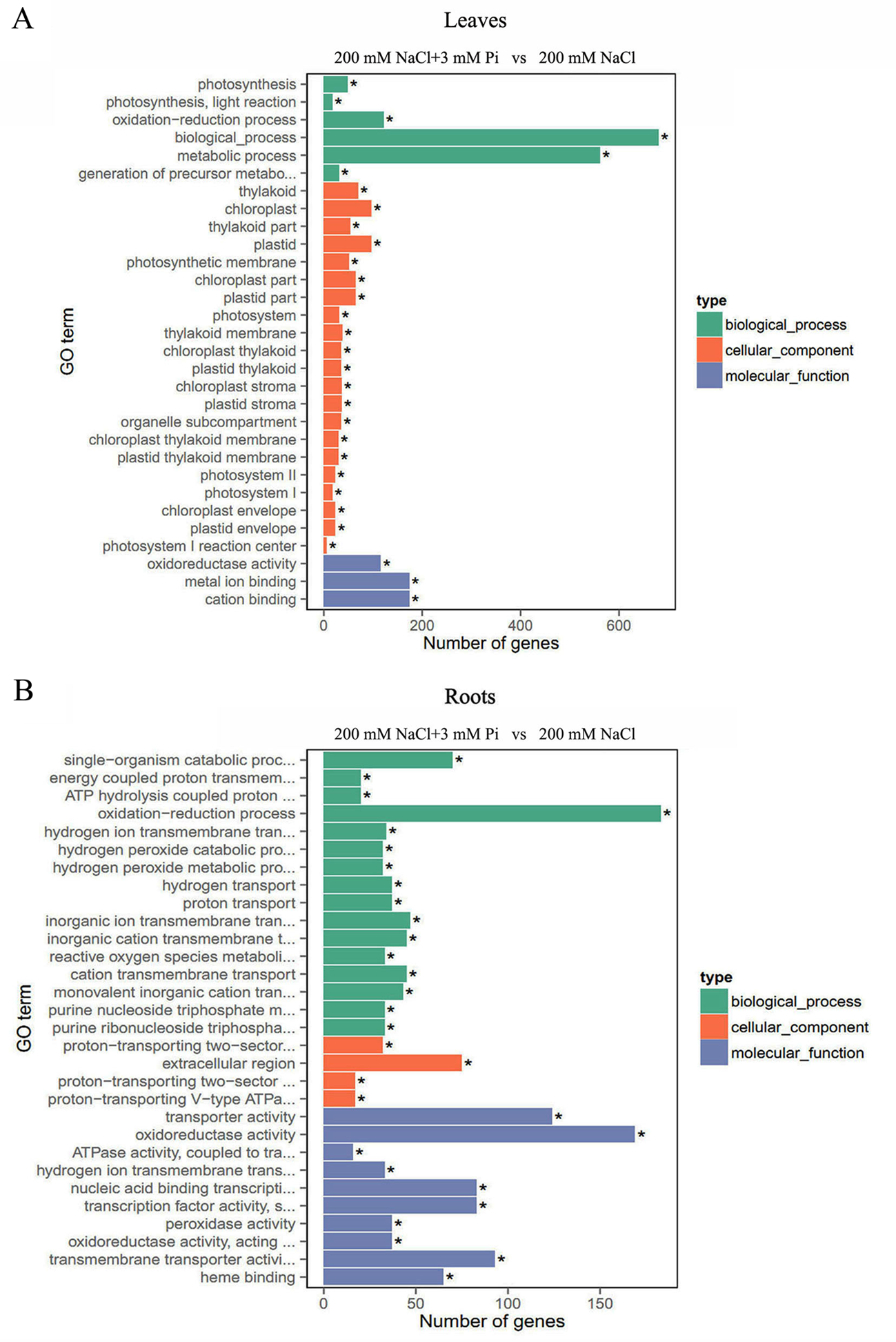
**

**Figure S8** Kyoto Encyclopedia of Genes and Genomes (KEGG) pathways of DEGs based on KEGG enrichment analysis in leaves (A) and roots (B) between 200 mM NaCl and 200 mM NaCl+3 mM Pi group.

**
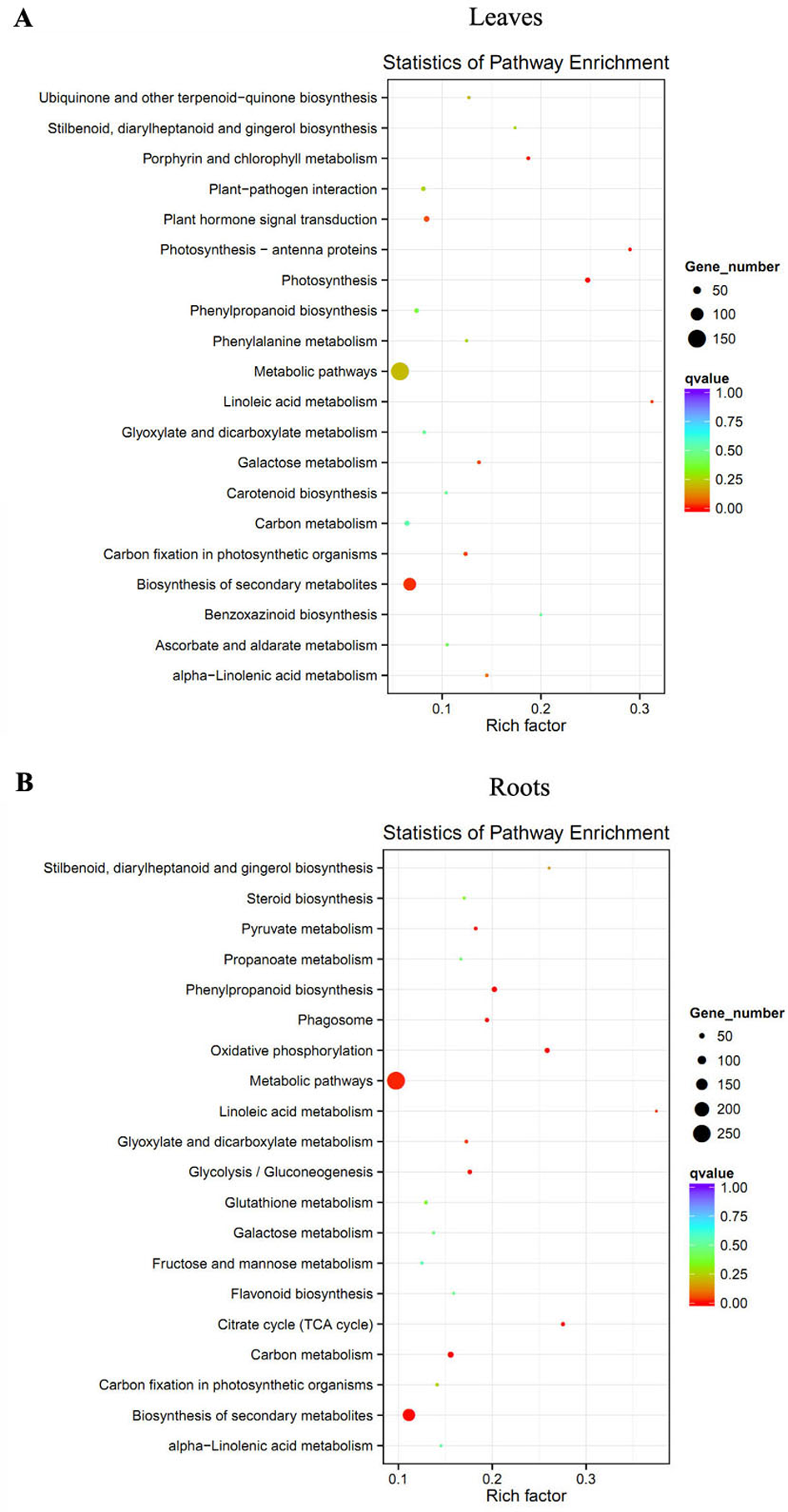
**

**Figure S9** Distribution of differentially expressed genes (DEPs) up-regulated or down-regulated in leaves and roots in 200 mM NaCl group compared with in 200 mM NaCl+3 mM Pi group.

**
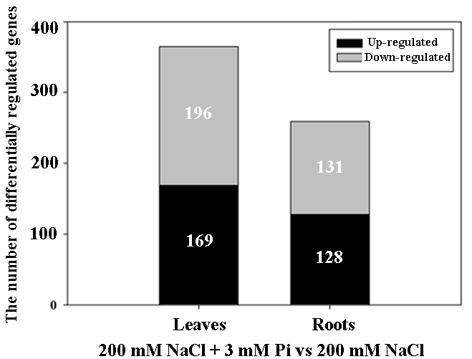
**

**Figure S10** Gene ontology categories of DEPs based on GO enrichment analysis in leaves (A) and roots (B) between 200 mM NaCl and 200 mM NaCl+3 mM Pi group.

**
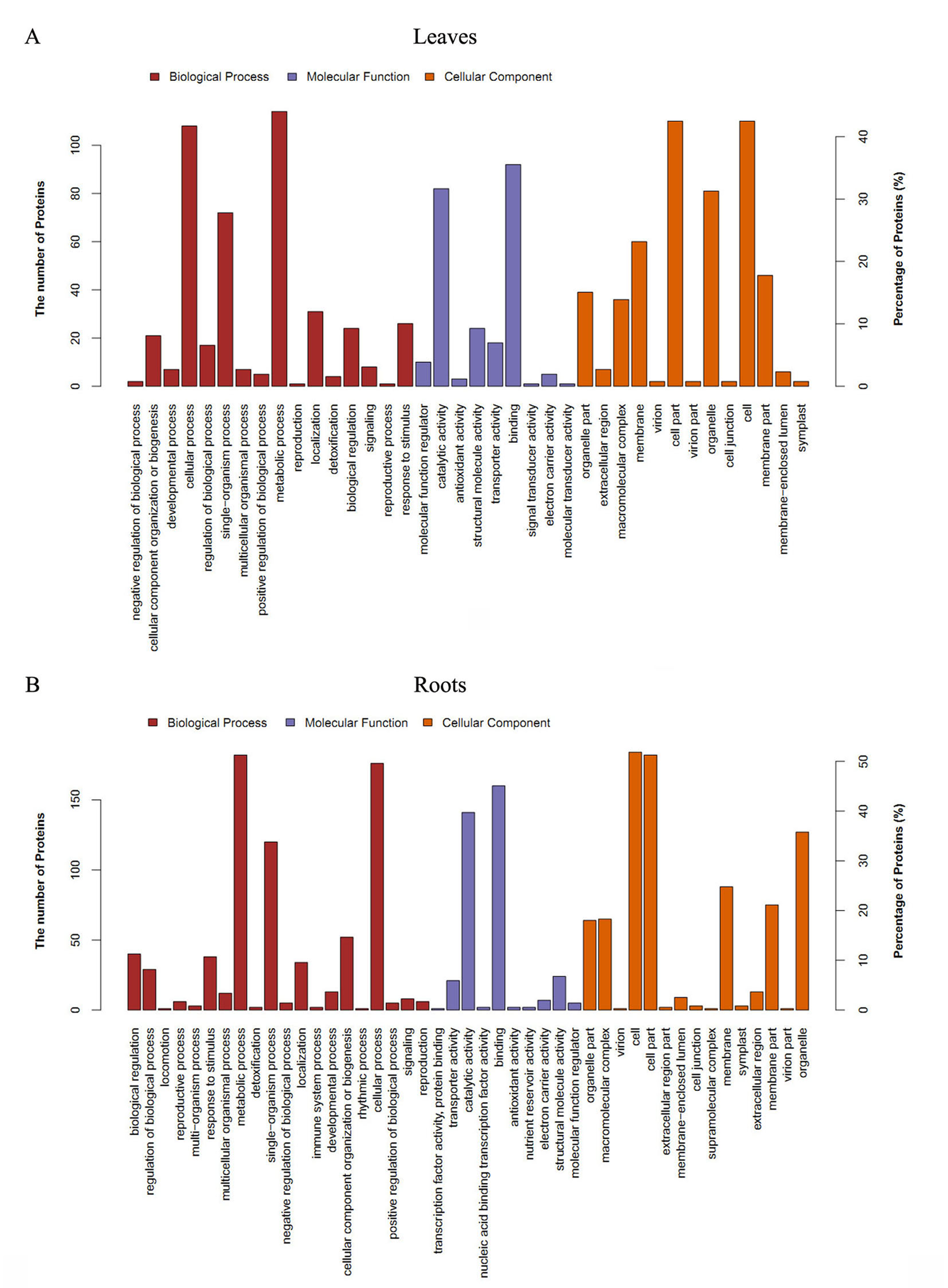
**

**Figure S11** KEGG pathways of DEPs based on KEGG enrichment analysis in leaves (A) and roots (B) between 200 mM NaCl and 200 mM NaCl+3 mM Pi group.

**
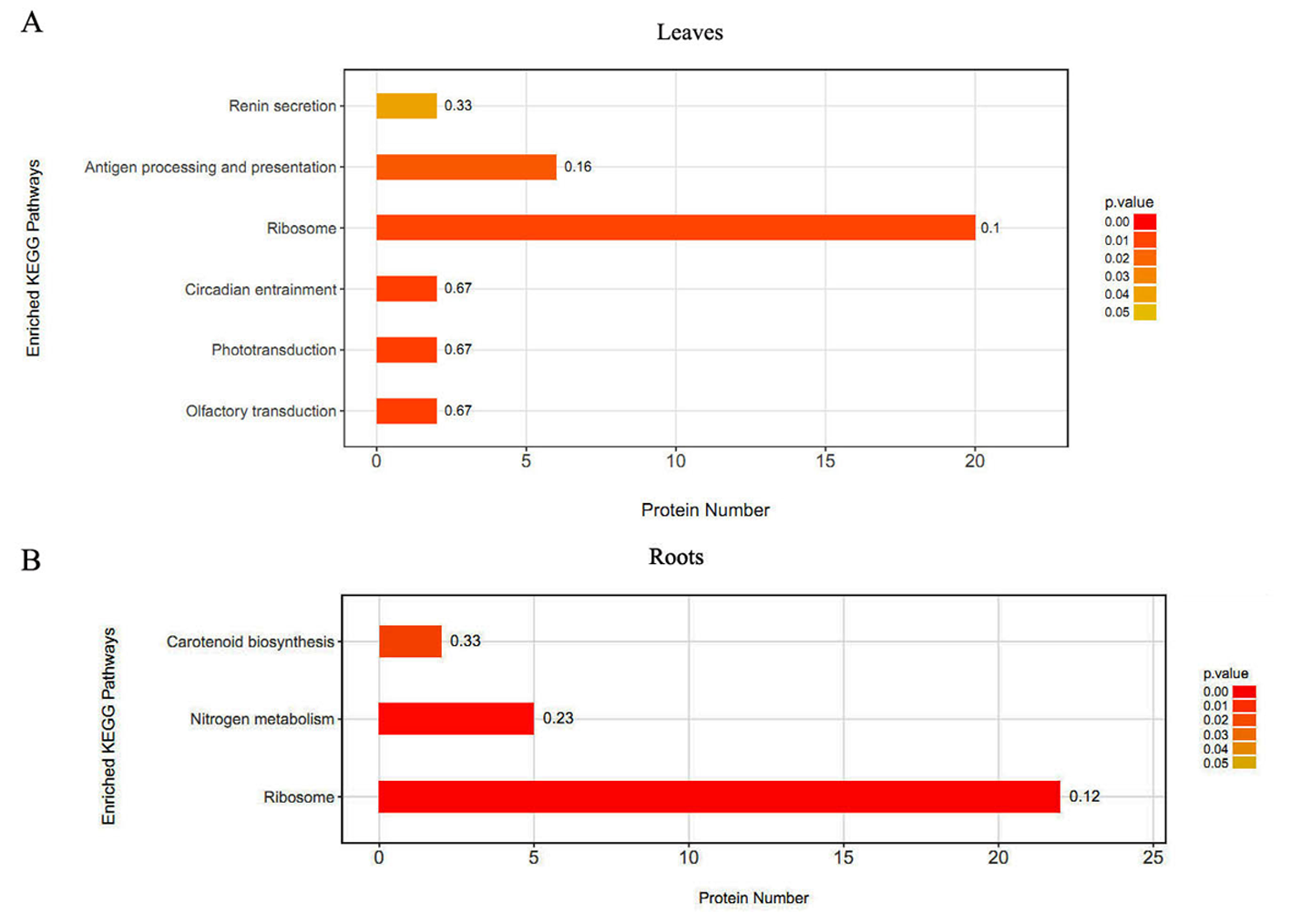
**

**Table S1**  Primers used in quantitative real-time PCR.

| Gene name | Genbank accession number | Forward/reverse primer | Sequence (5’-3’) | Product  (bp) |
| --- | --- | --- | --- | --- |
| *18S rRNA* | AF168884 | Forward | CCATCCCTCCGTAGTTAGCTTCT | 151 bp |
|  |  | Reverse | CCTGTCGGCCAAGGCTATATAC |  |
|  |  |  |  |  |
| *ZmPHR1* | JF831533 | Forward | CACCCTTTATTTCTCAGTCATCCAA | 205 bp |
|  |  | Reverse | TCATTTTGTGTAGCACTCTCATCAG |  |
|  |  |  |  |  |
| *ZmPhytase2* | AJ223471 | Forward | CGGGCAACCTGGCGTGG | 134 bp |
|  |  | Reverse | CGCCTTGGTGACAGCCGC |  |
|  |  |  |  |  |
| *ZmPHT1;3* | GRMZM2G112377 | Forward | CGTCCTCATCCCTCGCTGC | 93 bp |
|  |  | Reverse | CCCCGCCTCAACCTGTCTC |  |
|  |  |  |  |  |
| *ZmPHT1;4* | GRMZM2G170208 | Forward | TGGCGTGTTTGCCTTCGTT | 136 bp |
|  |  | Reverse | CTCCTCCAGTGCCTTCCCCT |  |
|  |  |  |  |  |
| *ZmPHT1;8* | GRMZM2G045473 | Forward | CATTGTCGGGTCATTTGGGTTC | 207 bp |
|  |  | Reverse | GCCTCCTCGTCGTTCTCGC |  |
|  |  |  |  |  |
| *ZmPHT1; 9* | GRMZM2G154090 | Forward | ACGACCACTTCAACTCCACCG | 157 bp |
|  |  | Reverse | GCCTGCTTGGCGTTCTTGG |  |
|  |  |  |  |  |
| *ZmNHX1* | AY270036 | Forward | ATGTGGCGTTACGGTGCGG | 206 bp |
|  |  | Reverse | GGTCATTTGGTGGGCGGG |  |
|  |  |  |  |  |
| *ZmHAK1* | GRMZM2G093826 | Forward | CGTGCCCACCAACCAGATGAC | 123 bp |
|  |  | Reverse | TTGATGAGTCGGGGGAACACG |  |
|  |  |  |  |  |
| *ZmP5CS* | DQ864376 | Forward | GCGAGGAAGTGGGCAAGTGGT | 250 bp |
|  |  | Reverse | TTGGGGAGGTGGGGTGGC |  |
|  |  |  |  |  |
| *ZmP5CR* | DQ026301 | Forward | CCAGCCTGTGCCAACCGC | 147 bp |
|  |  | Reverse | GTGCGGATGGCGGAGGC |  |
|  |  |  |  |  |
| *ZmTPS1* | AF529266 | Forward | GGTTGCAGCGTTTCCTATTG | 177 bp |
|  |  | Reverse | AATCAAGAGATCGGTCCAGATG |  |
|  |  |  |  |  |
| *ZmSOD4* | XM_008650839 | Forward | TAAGCACCTGTGGCAACCGAT | 115 bp |
|  |  | Reverse | ACGAAACGGTCGGAATGCC |  |

**Table S2** The differentially expressed genes (DEGs) identified between the 200 mM NaCl group and the 200 mM NaCl+3 mM Pi group by the transcriptomic analysis (the fold change is |log_2_(200 mM NaCl+3 mM Pi /200 mM NaCl)| >1.0, and P value < 0.05).

| Tissue | GeneID | Gene description | Fold change P value | |
| --- | --- | --- | --- | --- |
| **Leaves Metabolic pathways** | |  |  |  |
|  | *Zm00001d051194* | Arginine decarboxylase | 4.480 | 2.56E-11 |
|  | *Zm00001d053015* | Fructose-bisphosphate aldolase | 2.256 | 1.33E-12 |
|  | *Zm00001d002589* | Beta-carotene hydroxylase | 2.213 | 1.53E-08 |
|  | *Zm00001d039258* | Triose phosphate/phosphate translocator, chloroplastic | 1.753 | 1.46E-44 |
|  | *Zm00001d035001* | Chloroplast ferredoxin 1 | 1.600 | 4.01E-78 |
|  | *Zm00001d014083* | Beta-amylase | 1.451 | 3.18E-05 |
|  | *Zm00001d043299* | Photosystem II reaction center W protein | 1.404 | 1.06E-179 |
|  | *Zm00001d018157* | Chlorophyll a-b binding protein, chloroplastic | 1.390 | 9.20E-16 |
|  | *Zm00001d041819* | Photosystem I reaction center subunit N | 1.344 | 2.72E-182 |
|  | *Zm00001d045575* | Ferredoxin | 1.060 | 5.77E-09 |
|  | *Zm00001d032332* | Quinone oxidoreductase-like protein At1g23740 | 1.009 | 0.000116 |
|  | *Zm00001d019850* | Fatty acyl-CoA reductase | -1.246 | 2.02E-05 |
|  | *Zm00001d044159* | Cytochrome P450 monooxygenase CYP72A5 | -1.469 | 1.86E-05 |
|  | *Zm00001d011845* | NADPH quinone oxidoreductase 2 | -1.537 | 9.37E-14 |
|  | *Zm00001d026121* | Amino acid transport protein | -1.584 | 1.76E-07 |
|  | *Zm00001d042453* | Fructose-bisphosphate aldolase | -1.807 | 6.50E-28 |
|  | *Zm00001d005823* | Flavonoid 3-monooxygenase | -1.858 | 2.55E-05 |
|  | *Zm00001d028759* | Pyruvate decarboxylase | -2.137 | 3.92E-09 |
|  | *Zm00001d026156* | Putative glycerol 3-phosphate permease | -2.249 | 2.24E-14 |
|  | *Zm00001d023941* | Carbohydrate transporter/ sugar porter | -2.273 | 8.61E-14 |
|  | *Zm00001d017425* | Cytochrome b5 | -2.629 | 1.31E-06 |
|  | *Zm00001d017984* | Hexosyltransferase | -3.364 | 1.87E-12 |
|  | *Zm00001d011734* | Phosphatase phosphol | -3.662 | 2.76E-23 |
|  | *Zm00001d039094* | 3-ketoacyl-CoA synthase | -3.913 | 5.02E-06 |
|  | *Zm00001d021168* | Glycosyltransferase | -5.992 | 4.40E-09 |
|  | **Plant growth** |  |  |  |
|  | *Zm00001d043047* | Alpha-expansin 1 | 6.128 | 8.86E-05 |
|  | *Zm00001d047096* | Beta-expansin 1a | 5.323 | 7.05E-07 |
|  | *Zm00001d026160* | Cortical cell-delineating protein | 2.420 | 1.11E-34 |
|  | *Zm00001d030786* | Bundle sheath defective protein 2 | 1.594 | 2.47E-05 |
|  | *Zm00001d003867* | Extensin | 1.015 | 9.28E-06 |
|  | *Zm00001d050899* | Cell number regulator 2 | -1.253 | 0.000114 |
|  | *Zm00001d012900* | Cell number regulator 10 | -1.513 | 9.19E-10 |
|  | *Zm00001d013465* | Dwarf plant9 | -1.543 | 4.74E-13 |
|  | **Transcription factors** |  |  |  |
|  | *Zm00001d011515* | MYB-related transcription factor | 5.542 | 7.77E-08 |
|  | *Zm00001d002754* | HB transcription factor | 4.307 | 8.11E-08 |
|  | *Zm00001d023336* | Putative WRKY DNA-binding domain protein | 1.596 | 3.81E-11 |
|  | *Zm00001d041604* | BZIP transcription factor | -1.125 | 9.02E-05 |
|  | *Zm00001d008793* | Putative WRKY DNA-binding domain protein | -3.355 | 0.000114 |
|  | *Zm00001d024324* | AP2-EREBP transcription factor | -3.587 | 1.01E-06 |
|  | *Zm00001d043706* | BHLH transcription factor | -3.869 | 1.89E-28 |
|  | *Zm00001d019207* | NAC protein | -4.029 | 7.39E-23 |
|  | **Translation and replication** | |  |  |
|  | *Zm00001d027442* | 50S ribosomal protein L6 | 2.046 | 4.29E-09 |
|  | *Zm00001d052216* | 50S ribosomal protein L29 | 1.876 | 2.04E-18 |
|  | *Zm00001d019898* | Plastid-specific 30S ribosomal protein 2 | 1.665 | 5.45E-09 |
|  | *Zm00001d049694* | Ribosome recycling factor | 1.632 | 3.49E-06 |
|  | *Zm00001d007012* | Ribonucleoprotein A | 1.589 | 1.05E-05 |
|  | *Zm00001d049160* | RNA polymerase sigma factor | 1.193 | 4.97E-05 |
|  | *Zm00001d021788* | Elongation factor 1-alpha | -1.631 | 2.08E-84 |
|  | *Zm00001d028139* | Nucleic acid binding protein | -2.149 | 1.07E-05 |
|  | **Transporters** |  |  |  |
|  | *Zm00001d011013* | Calcium pump1 | -1.118 | 8.41E-05 |
|  | *Zm00001d025831* | Ammonium transporter | -2.818 | 2.14E-05 |
|  | **Metal ion binding** |  |  |  |
|  | *Zm00001d023536* | Mg-protoporphyrin IX chelatase | 1.072 | 9.69E-08 |
|  | *Zm00001d029720* | ABC-type Co^2+^ transport system, permease component | 1.282 | 3.09E-05 |
|  | *Zm00001d021515* | Electron carrier/ transporter/ iron ion binding protein | 1.299 | 3.99E-22 |
|  | *Zm00001d048451* | Selenium-binding protein-like protein | 1.094 | 8.30E-05 |
|  | *Zm00001d047492* | Putative heavy metal transport/detoxification protein | -1.035 | 6.96E-11 |
|  | *Zm00001d019936* | Heavy metal-associated domain containing protein | -1.240 | 7.36E-06 |
|  | *Zm00001d032873* | Metal ion binding protein | -2.286 | 1.95E-266 |
|  | **Redox homeostasis** |  |  |  |
|  | *Zm00001d022456* | Peroxidase | 1.516 | 9.02E-06 |
|  | *Zm00001d031908* | Superoxide dismutase [Cu-Zn] | 1.243 | 3.77E-15 |
|  | *Zm00001d011154* | Glutathione S-transferase GST 27 | 1.108 | 6.82E-21 |
|  | *Zm00001d037079* | Glutathione peroxidase | 1.048 | 1.43E-05 |
|  | *Zm00001d054044* | Catalase | -1.080 | 4.01E-09 |
|  | *Zm00001d014341* | Peroxidase | -1.105 | 7.04E-08 |
|  | *Zm00001d020780* | Glutathione transferase GST 23 | -2.158 | 4.36E-09 |
|  | **Stress response** |  |  |  |
|  | *Zm00001d017288* | Aquaporin PIP2-4 | 2.332 | 5.02E-16 |
|  | *Zm00001d054093* | Senescence-associated protein DIN1 | 1.474 | 2.30E-06 |
|  | *Zm00001d022464* | Ultraviolet-B-repressible protein | 1.249 | 1.02E-105 |
|  | *Zm00001d024903* | Putative heat shock protein 90 family protein | 1.405 | 6.64E-09 |
|  | *Zm00001d052194* | Heat shock 22 kDa protein | 1.389 | 5.59E-19 |
|  | *Zm00001d004443* | Mannitol dehydrogenase | -1.013 | 1.53E-07 |
|  | *Zm00001d039037* | Mitogen-activated protein kinase | -1.136 | 1.64E-12 |
|  | *Zm00001d045064* | Putative MAP kinase family protein | -1.146 | 4.00E-05 |
|  | *Zm00001d044826* | Aquaporin NIP2-3 | -1.316 | 1.66E-14 |
|  | *Zm00001d029371* | Trehalose 6-phosphate phosphatase | -1.577 | 0.000145 |
|  | *Zm00001d047955* | Stress inducible protein coi6.1 | -1.621 | 6.22E-17 |
|  | *Zm00001d024778* | NaCl stress protein1 | -1.624 | 2.78E-25 |
|  | *Zm00001d028711* | Mitogen-activated protein kinase | -1.748 | 1.18E-06 |
|  | *Zm00001d003068* | Calcium-dependent protein kinase 2 | -1.876 | 1.70E-13 |
|  | *Zm00001d020552* | Senescence associated protein | -2.024 | 3.61E-26 |
|  | *Zm00001d006211* | Senescence-inducible chloroplast stay-green protein 1 | -2.516 | 1.28E-10 |
|  | *Zm00001d003981* | Drought-induced protein 1 | -2.971 | 4.49E-61 |
|  | *Zm00001d002623* | Protein DETOXIFICATION | -3.002 | 3.34E-05 |
|  | *Zm00001d011656* | Putative MAPKKK family protein kinase | -3.259 | 2.45E-05 |
|  | **Phytohormone regulation** | |  |  |
|  | *Zm00001d020732* | Gibberellin receptor GID1L2 | 1.978 | 1.20E-06 |
|  | *Zm00001d018973* | Auxin-responsive protein | -1.083 | 0.000157 |
|  | *Zm00001d023664* | ABA-responsive protein | -1.180 | 3.87E-10 |
|  | *Zm00001d043083* | Putative auxin efflux carrier-like protein PINY | -1.245 | 1.83E-05 |
|  | *Zm00001d001879* | Auxin response factor | -1.499 | 9.16E-11 |
|  | *Zm00001d044921* | Gibberellin receptor GID1L2 | -1.590 | 3.67E-14 |
|  | *Zm00001d035079* | Auxin regulated gene involved in organ size 5 | -2.161 | 1.56E-11 |
|  | *Zm00001d032046* | Cytokinin dehydrogenase 10 | -2.521 | 3.74E-18 |
|  | *Zm00001d000339* | Ethylene-responsive transcription factor 4 | -2.555 | 2.01E-06 |
|  |  |  |  |  |

| **Roots** | **Metabolic pathways** | |  |  |
| --- | --- | --- | --- | --- |
|  | *Zm00001d031959* | Fatty acyl-CoA reductase | 2.923 | 2.04E-31 |
|  | *Zm00001d008837* | Putative cytochrome P450 superfamily protein | 2.669 | 0.000156 |
|  | *Zm00001d046318* | Putative FAD-binding Berberine family protein | 2.551 | 1.68E-14 |
|  | *Zm00001d028931* | Hexosyltransferase | 2.514 | 1.12E-05 |
|  | *Zm00001d048925* | Glycosyltransferase | 2.086 | 1.46E-26 |
|  | *Zm00001d007789* | Serine/threonine-protein kinase | 2.025 | 2.94E-30 |
|  | *Zm00001d028260* | Glutamine synthetase | 2.005 | 2.44E-07 |
|  | *Zm00001d006119* | Ferredoxin | -1.328 | 0.0001627 |
|  | *Zm00001d016166* | Phosphoenolpyruvate carboxylase | -1.343 | 9.85E-11 |
|  | *Zm00001d053010* | V-type proton ATPase proteolipid subunit | -2.018 | 3.07E-43 |
|  | *Zm00001d005253* | Vacuolar ATP synthase subunit G | -2.049 | 9.21E-08 |
|  | *Zm00001d047705* | Cyclase/dehydrase family protein | -2.456 | 4.30E-14 |
|  | *Zm00001d023213* | Putative cytochrome P450 superfamily protein | -3.975 | 1.64E-70 |
|  | *Zm00001d043621* | Phosphatase phosphol | -5.798 | 1.49E-11 |
|  | **Plant growth** |  |  |  |
|  | *Zm00001d012899* | Cell number regulator 9 | 1.334 | 3.47E-05 |
|  | *Zm00001d050899* | Cell number regulator 2 | 1.325 | 1.17E-10 |
|  | *Zm00001d045420* | Root cap-specific protein | -3.518 | 3.15E-22 |
|  | *Zm00001d019560* | Beta-expansin 4 | -4.646 | 5.99E-18 |
|  | *Zm00001d047117* | Cortical cell-delineating protein | -8.571 | 3.47E-83 |
|  | *Zm00001d025018* | Alpha-expansin 3 | -9.408 | 3.79E-17 |
|  | **Transcription factors** | |  |  |
|  | *Zm00001d028999* | NAC transcription factor | 3.617 | 2.43E-08 |
|  | *Zm00001d053060* | MYB transcription factor | 3.039 | 1.71E-06 |
|  | *Zm00001d046751* | BZIP transcription factor | 2.534 | 8.93E-05 |
|  | *Zm00001d039764* | BHLH transcription factor | 2.493 | 4.13E-08 |
|  | *Zm00001d032040* | SRS transcription factor | 2.332 | 3.74E-06 |
|  | *Zm00001d043950* | WRKY transcription factor | 2.211 | 3.77E-16 |
|  | *Zm00001d042593* | AP2-EREBP transcription factor | 2.202 | 2.14E-45 |
|  | *Zm00001d029607* | GRAS transcription factor | 1.570 | 4.69E-06 |
|  | *Zm00001d020714* | HSF transcription factor | 1.405 | 1.42E-06 |
|  | *Zm00001d029855* | GRAS transcription factor | -1.059 | 0.000249 |
|  | *Zm00001d044004* | AP2-EREBP transcription factor | -1.070 | 9.06E-09 |
|  | *Zm00001d038270* | MYB transcription factor | -1.417 | 3.79E-06 |
|  | *Zm00001d047081* | C2C2-GATA transcription factor | -3.167 | 3.14E-27 |
|  | *Zm00001d018056* | BHLH transcription factor | -3.266 | 6.71E-29 |
|  | *Zm00001d039383* | BZIP transcription factor | -3.365 | 7.02E-07 |
|  | **Translation and replication** | |  |  |
|  | *Zm00001d026542* | G2-like transcription factor | 3.323 | 2.09E-05 |
|  | *Zm00001d026652* | DNA binding protein | 2.033 | 6.85E-05 |
|  | *Zm00001d051573* | Putative homeobox DNA-binding domain superfamily | 1.912 | 0.0003864 |
|  | *Zm00001d039495* | Nucleic acid binding protein | -1.127 | 3.18E-06 |
|  | *Zm00001d017726* | G2-like transcription factor | -1.866 | 0.0002135 |
|  | *Zm00001d051005* | DNA binding protein | -2.464 | 5.15E-05 |
|  | **Transporters** |  |  |  |
|  | *Zm00001d054060* | High affinity nitrate transporter | 5.864 | 3.75E-07 |
|  | *Zm00001d054057* | High affinity nitrate transporter | 4.883 | 0.000145 |
|  | *Zm00001d044717* | Potassium outward rectifying channel | 2.385 | 3.82E-07 |
|  | *Zm00001d049987* | Potassium transporter | 1.910 | 4.71E-11 |
|  | *Zm00001d034782* | Ammonium transporter | 1.737 | 1.74E-17 |
|  | **Metal ion binding** | |  |  |
|  | *Zm00001d044898* | Copper ion binding protein | 1.402 | 1.08E-05 |
|  | *Zm00001d048411* | Metal ion binding protein | -1.508 | 2.28E-13 |
|  | **Redox homeostasis** | |  |  |
|  | *Zm00001d034128* | Peroxidase | 6.625 | 6.28E-05 |
|  | *Zm00001d013212* | Peroxidase | 3.359 | 5.42E-07 |
|  | *Zm00001d005279* | Peroxidase | 2.268 | 1.95E-05 |
|  | *Zm00001d029706* | Glutathione S-transferase GSTU6 | 2.221 | 1.99E-05 |
|  | *Zm00001d054044* | Catalase | 1.927 | 8.15E-05 |
|  | *Zm00001d022453* | Peroxidase | 1.187 | 0.000112 |
|  | *Zm00001d021965* | Peroxidase | -1.179 | 4.72E-05 |
|  | *Zm00001d014366* | L-ascorbate oxidase | -1.373 | 2.30E-12 |
|  | *Zm00001d003707* | Peroxidase | -1.942 | 9.98E-21 |
|  | *Zm00001d027540* | Glutathione S-transferase | -1.991 | 1.52E-22 |
|  | *Zm00001d024281* | Polyamine oxidase | -2.062 | 1.23E-39 |
|  | *Zm00001d008173* | Peroxidase | -3.580 | 9.00E-66 |
|  | *Zm00001d049657* | Glutathione S-transferase GST 26 | -4.354 | 2.13E-11 |
|  | *Zm00001d038598* | Peroxidase | -5.868 | 4.94E-80 |
|  | **Stress response** |  |  |  |
|  | *Zm00001d044826* | Aquaporin NIP2-3 | 3.060 | 2.68E-09 |
|  | *Zm00001d026076* | Protein DETOXIFICATION | 2.485 | 3.28E-17 |
|  | *Zm00001d048178* | Polyol transporter protein 4 | 2.411 | 2.89E-06 |
|  | *Zm00001d028630* | Heat shock cognate 70 kDa protein 2 | 2.270 | 1.11E-31 |
|  | *Zm00001d017502* | Trehalose 6-phosphate phosphatase | 1.929 | 7.61E-24 |
|  | *Zm00001d037228* | Aquaporin NIP2-2 | 1.813 | 1.57E-05 |
|  | *Zm00001d023404* | Purple acid phosphatase | 1.740 | 1.59E-21 |
|  | *Zm00001d015914* | Bidirectional sugar transporter SWEET | 1.521 | 3.40E-05 |
|  | *Zm00001d011654* | Putative MAPKKK family protein kinase | 1.376 | 2.27E-20 |
|  | *Zm00001d019712* | Sucrose responsive element binding protein | 1.362 | 0.00031 |
|  | *Zm00001d003981* | Drought-induced protein 1 | 1.227 | 1.90E-25 |
|  | *Zm00001d018037* | Aquaporin NIP2-1 | 1.078 | 1.84E-07 |
|  | *Zm00001d022420* | Stress responsive protein | 1.058 | 2.37E-08 |
|  | *Zm00001d052194* | Heat shock 22 kDa protein | -1.099 | 7.23E-19 |
|  | *Zm00001d003006* | Aquaporin PIP2-5 | -1.460 | 5.14E-50 |
|  | *Zm00001d017288* | Aquaporin PIP2-4 | -1.688 | 9.16E-76 |
|  | *Zm00001d000222* | Bidirectional sugar transporter SWEET | -2.617 | 6.05E-09 |
|  | *Zm00001d004443* | Mannitol dehydrogenase | -2.758 | 8.24E-05 |
|  | *Zm00001d017485* | Aquaporin TIP2-2 | -4.598 | 2.44E-278 |
|  | **Phytohormone regulation** | |  | -4.598 |
|  | *Zm00001d024853* | Acc oxidase | 2.280 | 1.11E-08 |
|  | *Zm00001d023664* | ABA-responsive protein | 1.623 | 7.46E-30 |
|  | *Zm00001d000358* | Auxin response factor | 1.398 | 1.41E-08 |
|  | *Zm00001d018158* | Ethylene-responsive element binding protein 2 | 1.304 | 1.32E-10 |
|  | *Zm00001d001879* | Auxin response factor | -1.057 | 2.09E-06 |
|  | *Zm00001d018024* | Auxin efflux carrier component | -1.630 | 2.51E-13 |
|  | *Zm00001d051302* | SAUR11-auxin-responsive SAUR family member | -2.018 | 5.89E-06 |
|  | *Zm00001d041662* | Jasmonate-induced protein | -2.659 | 2.76E-15 |
|  | *Zm00001d033976* | Auxin-responsive protein | -3.227 | 3.05E-49 |

**Table S3. XLSX**

**Table S4 The differentially expressed proteins (DEPs) identified between the 200 mM NaCl group and the 200 mM NaCl+3 mM Pi group by the proteomic analysis (the ratio is |200Na+3Pi/200Na| > 1.2, and P value < 0.05).**

| Tissue | | Accession number Protein description Coverage Ratio P value | | | | | | | | | | | | |
| --- | --- | --- | --- | --- | --- | --- | --- | --- | --- | --- | --- | --- | --- | --- |
| **Leaves Metabolism** | | |  | | |  | | | |  | | |  | |
|  | | B6TT67 | | Glycosyltransferase | | 29.02 | | | | 1.528 | | | 5.71E-08 | |
|  | | P80680 | | Ferredoxin-thioredoxin reductase, variable chain | | 40.21 | | | | 1.440 | | | 3.09E-06 | |
|  | | Q19VG6 | | Major latex protein 22 | | 36.36 | | | | 1.387 | | | 2.90E-05 | |
|  | | B6U7P4 | | Lipid transfer protein1 | | 19.39 | | | | 1.384 | | | 3.34E-05 | |
|  | | B6ST66 | | Cytochrome P450 CYP92C5 | | 2.47 | | | | 1.343 | | | 0.00017 | |
|  | | B6SS02 | | Plastidic phosphate translocator-like protein1 | | 4.23 | | | | 1.322 | | | 0.00037 | |
|  | | A0A1D6KYX1 | | GTP binding protein2 | | 10.53 | | | | 1.285 | | | 0.00145 | |
|  | | Q6VWJ0 | | Caffeoyl-CoA 3-O-methyltransferase 1 | | 32.56 | | | | 1.251 | | | 0.0045 | |
|  | | B6UAB8 | | Lipid binding protein | | 23.79 | | | | 1.213 | | | 0.01467 | |
|  | | C4JAX7 | | UDP-sulfoquinovose synthase chloroplastic | | 48.73 | | | | 0.769 | | | 0.0007 | |
|  | | A0A1D6G8G7 | | Mannose-1-phosphate guanylyltransferase 1 | | 12.58 | | | | 0.783 | | | 0.00152 | |
|  | | E2IPC6 | | Chloroplast thioredoxin M-type 2 | | 30.41 | | | | 0.786 | | | 0.00186 | |
|  | | B4G1F0 | | ATP binding protein | | 22.22 | | | | 0.805 | | | 0.00498 | |
|  | | P69523 | | Cytochrome b559 subunit beta | | 20.51 | | | | 0.816 | | | 0.00841 | |
|  | | B6T927 | | NAD(P)H-quinone oxidoreductase subunit S | | 67.23 | | | | 0.827 | | | 0.01337 | |
|  | | P24993 | | Photosystem II reaction center protein H | | 41.1 | | | | 0.830 | | | 0.01541 | |
|  | | P60138 | | Photosystem II reaction center protein L | | 36.84 | | | | 0.831 | | | 0.01577 | |
|  | | **Plant growth** | |  | |  | | | |  | | |  | |
|  | | A0A077D360 | | Cellulose synthase | | | 5.61 | | 1.435 | | 3.93E-06 | | | |
|  | | Q94KT3 | | Alpha-expansin 5 | | | 6.19 | | 1.240 | | 0.00646 | | | |
|  | | B6TVC4 | | Cell envelope integrity inner membrane protein TolA | | | 31.99 | | 1.219 | | 0.01221 | | | |
|  | | B6SX43 | | Cortical cell-delineating protein | | | 74.68 | | 0.767 | | 0.00061 | | | |
|  | | A0A1D6ML43 | | Cell number regulator 8 | | | 50.93 | | 0.825 | | 0.01211 | | | |
|  | | **Transcriptional regulators** | | | | |  | |  | |  | | | |
|  | | B4FVF9 | | CCAAT-DR1 transcription factor | | | 6.11 | | 1.449 | | 2.08E-06 | | | |
|  | | A0A1D6KJN8 | | Transcription factor bHLH62 | | | 2.05 | | 0.826 | | 0.01276 | | | |
|  | | **Translation and replication** | | | | |  | |  | |  | | | |
|  | | B6TQN7 | | 60S acidic ribosomal protein P1 | | | 79.82 | | 1.451 | | 1.96E-06 | | | |
|  | | B6TA83 | | Replication factor C subunit 4 | | | 5.14 | | 1.431 | | 4.60E-06 | | | |
|  | | A0A1D6MPT4 | | H/ACA ribonucleoprotein complex subunit 4 | | | 20.73 | | 1.367 | | 6.65E-05 | | | |
|  | | B6T1F1 | | Ribosomal protein L19 | | | 31.88 | | 1.354 | | 0.00011 | | | |
|  | | B4FIA6 | | Histone H2A | | | 23.7 | | 1.330 | | 0.00028 | | | |
|  | | B4FSG3 | | 60S ribosomal protein L7-1 | | | 57.09 | | 1.309 | | 0.00061 | | | |
|  | | B6TUN0 | | DNA-binding protein S1FA2 | | | 24.32 | | 1.297 | | 0.00092 | | | |
|  | | A0A1D6HQL5 | | Nucleic acid binding | | | 4.62 | | 1.294 | | 0.00105 | | | |
|  | | A0A1D6Q118 | | Splicing factor PWI domain-containing protein | | | 2.17 | | 1.267 | | 0.00268 | | | |
|  | | B4FS20 | | Translation elongation factor EF1B | | | 8.39 | | 1.232 | | 0.00831 | | | |
|  | | B4G1Q5 | | 50S ribosomal protein L10 chloroplastic | | | 42.34 | | 1.210 | | 0.01596 | | | |
|  | | A0A1D6JTS7 | | Nuclear pore complex protein NUP155 | | | 3.51 | | 1.207 | | 0.01751 | | | |
|  | | A0A1D6L8D5 | | Elongation factor P | | | 4.62 | | 1.200 | | 0.02123 | | | |
|  | | B4G1R9 | | Glutamyl-tRNA(Gln) amidotransferase subunit C | | | 17.93 | | 0.766 | | 0.00058 | | | |
|  | | A0A1D6PWW9 | | AT-hook motif nuclear-localized protein 1 | | | 5.86 | | 0.771 | | 0.00075 | | | |
|  | | A0A1D6K5M3 | | Nuclear transcription factor Y subunit B-2 | | | 13.06 | | 0.806 | | 0.00518 | | | |
|  | | B4FLV6 | | Protein translation factor SUI1 | | | 44.35 | | 0.809 | | 0.00607 | | | |
|  | | B6SW27 | | Bifunctional aminoacyl-tRNA synthetase | | | 3.17 | | 0.819 | | 0.00965 | | | |
|  | | A0A1D6HFV5 | | Translation initiation factor IF-2 chloroplastic | | | 2.14 | | 0.824 | | 0.01201 | | | |
|  | | B6T3F2 | | Histone H2A | | | 24.26 | | 0.830 | | 0.01526 | | | |
|  | | **Transporters** | |  | | |  | |  | |  | | | |
|  | | C0P6N0 | | Calcium load-activated calcium channel | | | 9.55 | | 1.589 | | 2.88E-09 | | | |
|  | | A0A1D6HPY9 | | ABC transporter F family member 3 | | | 3.06 | | 1.265 | | 0.00287 | | | |
|  | | A0A096PXB4 | | Vacuolar cation/proton exchanger 3 | | | 1.3 | | 0.632 | | 3.78E-09 | | | |
|  | | A0A1D6HHV5 | | Cation/H(+) antiporter 1 | | | 1.01 | | 0.726 | | 3.70E-05 | | | |
|  | | A0A1D6DSW6 | | K(+) efflux antiporter 2 chloroplastic | | | 31.72 | | 0.748 | | 0.00018 | | | |
|  | | B6SJM6 | | Copper transporter 1 | | | 4.88 | | 0.753 | | 0.00025 | | | |
|  | | Q6GUH9 | | Phosphate transport protein | | | 14.47 | | 0.772 | | 0.00084 | | | |
|  | | C0PHL2 | | Monosaccharide transporter1 | | | 8.41 | | 0.788 | | 0.00204 | | | |
|  | | A0A1D6MQM6 | | Carbohydrate transporter/ sugar porter | | | 2.23 | | 0.809 | | 0.00598 | | | |
|  | | A0A1D6JIE4 | | K(+) efflux antiporter 2 chloroplastic | | | 11.81 | | 0.813 | | 0.00726 | | | |
|  | | **Metal ion binding** | | | | |  | |  | |  | | | |
|  | | A0A1D6FV33 | | Metal tolerance protein A2 | | | 2.51 | | 1.339 | | 0.0002 | | | |
|  | | A0A1D6H6V1 | | Calcium-binding EF hand family protein | | 2.77 | | 1.228 | | | | 0.00925 | |  |
|  | | Q43712 | | Calcium-binding protein | | 53.92 | | 1.216 | | | | 0.0132 | |  |
|  | | B6TG98 | | Copper ion binding protein | | 16.99 | | 1.207 | | | | 0.01733 | |  |
|  | | A0A1D6LGF5 | | Metal tolerance protein A2 | | 2.46 | | 0.804 | | | | 0.0046 | |  |
|  | | **Stress response** | |  | |  | |  | | | |  | |  |
|  | | B6TIP9 | | 17.5 kDa class II heat shock protein | | 24.68 | | 1.622 | | | | 5.60E-10 | |  |
|  | | B4G197 | | 16.9 kDa class I heat shock protein 1 | | 10.53 | | 1.506 | | | | 1.61E-07 | |  |
|  | | B4F9E8 | | 17.4 kDa class III heat shock protein | | 30.99 | | 1.331 | | | | 0.00027 | |  |
|  | | A0A1D6M4E1 | | Glutathione transferase41 | | 8.62 | | 1.303 | | | | 0.00076 | |  |
|  | | B4F9K4 | | 17.5 kDa class II heat shock protein | | 30.91 | | 1.286 | | | | 0.00136 | |  |
|  | | Q5EBY7 | | Heat shock protein 70 | | 58.18 | | 1.268 | | | | 0.0026 | |  |
|  | | A0A1D6HR96 | | Purple acid phosphatase | | 1.83 | | 1.205 | | | | 0.01818 | |  |
|  | | B6T9C5 | | Senescence-associated protein | | 16.33 | | 0.730 | | | | 4.90E-05 | |  |
|  | | A0A1D6GRK5 | | Aquaporin PIP2-2 | | 3.89 | | 0.744 | | | | 0.00014 | |  |
|  | | K7V8K5 | | Peroxidase | | 24.02 | | 0.787 | | | | 0.00197 | |  |
|  | | B4FA21 | | Purple acid phosphatase | | 29.71 | | 0.793 | | | | 0.0026 | |  |
|  | | A0A1D6GUG0 | | Peroxidase | | 3.79 | | 0.812 | | | | 0.00698 | |  |
|  | | Q9FQA3 | | Glutathione transferase GST 23 | | 6.31 | | 0.823 | | | | 0.01148 | |  |
|  | | B6TMF3 | | Wound/stress protein | | 29.41 | | 0.832 | | | | 0.01645 | |  |
|  | | **Phytohormone regulation** | | | |  | |  | | | |  | |  |
|  | | A0A1D6GKQ2 | | ARF guanine-nucleotide exchange factor GNOM | | 1.27 | | 1.243 | | | | 0.00586 | |  |
|  | | A0A1D6EB22 | | Abscisic acid stress ripening3 | | 63.94 | | 0.529 | | | | 3.62E-16 | |  |
|  | | A0A1D6KQ78 | | Auxin-responsive family protein | | 5.79 | | 0.812 | | | | 0.00698 | |  |
|  | |  | |  | |  | |  | | | |  | |  |
| **Roots** | **Metabolism** | | |  |  | | | | |  | | |  | |
|  | A0A1D6M542 | | | Ferredoxin | 6.49 | | | | | 2.424 | | | 0.011866 | |
|  | A0A1D6HL75 | | | Ferredoxin--nitrite reductase chloroplastic | 40 | | | | | 1.541 | | | 0.026054 | |
|  | A0A1D6PZA5 | | | Nitrate reductase | 2.81 | | | | | 1.488 | | | 0.037925 | |
|  | B4FCQ9 | | | Ubiquitin-conjugating enzyme E2 30 | 12.16 | | | | | 1.453 | | | 0.004209 | |
|  | A0A1D6K343 | | | Beta-amylase | 2.45 | | | | | 1.398 | | | 0.038175 | |
|  | A0A1D6F844 | | | NAD(P)-binding Rossmann-fold superfamily | 40.61 | | | | | 1.303 | | | 0.039564 | |
|  | A0A1D6K0R8 | | | Chloroplast protein HCF243 | 2.17 | | | | | 1.238 | | | 0.038225 | |
|  | A0A1D6K902 | | | Pyruvate kinase | 20.37 | | | | | 1.235 | | | 0.030087 | |
|  | A0A1D6NLZ8 | | | Cytochrome c oxidase polypeptide Vb | 87.5 | | | | | 1.223 | | | 0.030349 | |
|  | A0A1D6PN40 | | | Putative cytochrome P450 superfamily | 3.31 | | | | | 1.207 | | | 0.010258 | |
|  | A0A1D6DU94 | | | Ubiquitin-conjugating enzyme E2 11 | 38.66 | | | | | 0.395 | | | 0.007819 | |
|  | B6SKR4 | | | Cytochrome c | 39.29 | | | | | 0.607 | | | 0.038616 | |
|  | C4JAX7 | | | UDP-sulfoquinovose synthase chloroplastic | 37.29 | | | | | 0.609 | | | 0.023379 | |
|  | Q0GR41 | | | NADH dehydrogenase subunit 2 | 6.35 | | | | | 0.729 | | | 0.028674 | |
|  | A0A1D6FIR4 | | | V-type proton ATPase proteolipid subunit | 24.19 | | | | | 0.745 | | | 0.000943 | |
|  | C0PJV3 | | | Mannose-1-phosphate guanylyltransferase 1 | 13.73 | | | | | 0.823 | | | 0.035877 | |
|  | C0HF31 | | | Adenine nucleotide transporter BT1 | 3.81 | | | | | 0.829 | | | 0.002063 | |
|  | **Plant growth** | | |  |  | | | | |  | | |  | |
|  | A0A1D6GQ42 | | | Cell division cycle protein 48 | 55.49 | | | | | 1.220 | | | 0.005278 | |
|  | B6UG74 | | | Cortical cell-delineating protein | 28.24 | | | | | 0.678 | | | 0.026089 | |
|  | A0A1D6PJI4 | | | Expansin-like A2 | 4.86 | | | | | 0.762 | | | 0.037055 | |
|  | K7UEH4 | | | Cell division control protein 48 homolog D | 6.71 | | | | | 0.809 | | | 0.008207 | |
|  | **Transcriptional regulators** | | | |  | | | | |  | | |  | |
|  | C0PLN4 | | | DBP transcription factor | 2.86 | | | | | 1.267 | | | 0.001339 | |
|  | B4G1E5 | | | Cytosolic Fe-S cluster assembly factor NBP35 | 8.05 | | | | | 1.243 | | | 0.047842 | |
|  | **Translation and replication** | | | |  | | | | |  | | |  | |
|  | B4FW18 | | | 40S ribosomal protein S28 | 40 | | | | | 1.541 | | | 0.026054 | |
|  | B6T3L4 | | | Ribosomal protein L9 | 7.88 | | | | | 1.527 | | | 0.036976 | |
|  | B6T361 | | | 60S acidic ribosomal protein P1 | 75.23 | | | | | 1.502 | | | 0.039628 | |
|  | A0A1D6GFP8 | | | 40S ribosomal protein S18 | 48.46 | | | | | 1.393 | | | 0.001616 | |
|  | B6SGI4 | | | Ribosomal protein L37 | 36.17 | | | | | 1.373 | | | 0.036856 | |
|  | B6T595 | | | 60S ribosomal protein L17 | 42.11 | | | | | 1.350 | | | 0.013995 | |
|  | A0A1D6F4G1 | | | Eukaryotic translation initiation factor 2 | 15.53 | | | | | 1.293 | | | 0.040812 | |
|  | B4FBD6 | | | Ribonuclease 1 | 26.64 | | | | | 0.823 | | | 0.043102 | |
|  | B4FGA5 | | | Putative small nuclear ribonucleoprotein G | 41.25 | | | | | 0.823 | | | 0.047381 | |
|  | A0A1D6JWT7 | | | B-block binding subunit of TFIIIC | 1.47 | | | | | 0.726 | | | 0.040753 | |
|  | Q6R9F9 | | | DNA-directed RNA polymerase | 0.95 | | | | | 0.663 | | | 0.000795 | |
|  | **Transporters** | | |  |  | | | | |  | | |  | |
|  | A0A1D6HC37 | | | High affinity nitrate transporter | 23.15 | | | | | 1.626 | | | 0.011126 | |
|  | A6QKV9 | | | Putative high affinity nitrate transporter | 11.83 | | | | | 1.409 | | | 0.034144 | |
|  | B6TGS2 | | | Calcium load-activated calcium channel | 14.43 | | | | | 1.344 | | | 0.004091 | |
|  | K7TK04 | | | Carbohydrate transporter/ sugar porter | 9 | | | | | 0.573 | | | 0.00126 | |
|  | C4JC09 | | | Phosphate transporter protein 9 | 17.37 | | | | | 0.660 | | | 0.046708 | |
|  | A0A1D6KXB5 | | | Organic cation/carnitine transporter 7 | 4.4 | | | | | 0.762 | | | 0.02335 | |
|  | Q6I681 | | | Ascorbatetransmembrane electron transporter 1 | 14.41 | | | | | 0.796 | | | 0.017527 | |
|  | **Metal ion binding** | | | |  | | | | |  | | |  | |
|  | B4FGG7 | | | Calcium ion binding protein | 13.72 | | | | | 0.741 | | | 0.027441 | |
|  | **Stress response** | | |  |  | | | | |  | | |  | |
|  | A0A1D6DVY9 | | | Peroxidase | 2.63 | | | | | 1.490 | | | 0.003313 | |
|  | A0A1D6L1Z7 | | | Glutamine synthetase | 15.38 | | | | | 1.407 | | | 0.003489 | |
|  | A0A1D6FN98 | | | Heat shock 70 kDa protein 14 | 34.45 | | | | | 1.310 | | | 0.005246 | |
|  | B6U3T7 | | | Aquaporin TIP2.1 | 3.63 | | | | | 0.704 | | | 0.043076 | |
|  | A0A1D6IWU4 | | | Purple acid phosphatase | 7.61 | | | | | 0.730 | | | 0.049142 | |
|  | B6TMF3 | | | Wound/stress protein | 26.47 | | | | | 0.751 | | | 0.01053 | |
|  | Q9FQC7 | | | Glutathione S-transferase | 48.89 | | | | | 0.765 | | | 0.049712 | |
|  | A0A1D6ELH9 | | | Peroxidase | 25.67 | | | | | 0.815 | | | 0.042059 | |

**Table S5.XLSX**
